# Defining low-risk high hyperdiploidy in patients with paediatric acute lymphoblastic leukaemia: a retrospective analysis of data from the UKALL97/99 and UKALL2003 clinical trials

**DOI:** 10.1016/S2352-3026(21)00304-5

**Published:** 2021-10-26

**Authors:** Amir Enshaei, Ajay Vora, Christine J Harrison, John Moppett, Anthony V Moorman

**Affiliations:** a Leukaemia Research Cytogenetics Group, Wolfson Childhood Cancer Research Centre, Clinical and Translational Research Institute, Newcastle University, Newcastle-upon-Tyne, UK; b Department of Haematology, Great Ormond Street Hospital, London, UK; c Department of Haematology, Bristol Royal Hospital for Children, Bristol, UK

## Abstract

**Background:**

High hyperdiploidy is the most common genetic subtype of childhood acute lymphoblastic leukaemia and is associated with a good outcome. However, some patients relapse and, given its prevalence, patients with high hyperdiploidy account for a large proportion of all relapses. We aimed to evaluate putative risk factors and determine the optimal pattern of trisomies for predicting outcome.

**Methods:**

We used discovery and validation cohorts from consecutive trials—UKALL97/99 (n=456) and UKALL2003 (n=725)—to develop the prognostic profile. UKALL97/99 recruited patients aged 1–18 years between Jan 1, 1997, and June 15, 2002, and UKALL2003 recruited children and young adults aged 1–24 years between Oct 1, 2003, and June 30, 2001, from the UK and Ireland who were newly diagnosed with acute lymphoblastic leukaemia. Cytogenetic and fluorescence in-situ hybridisation testing was performed on pre-treatment bone marrow samples by regional UK National Health Service genetic laboratories or centrally by the Leukaemia Research Cytogenetics Group, and results were reported using established nomenclature and definitions. We examined the prognostic effect of previously proposed genetic and non-genetic risk factors among patients with high hyperdiploid acute lymphoblastic leukaemia treated on UKALL2003. We used Bayesian information criterion, targeted projection pursuit, and multivariate analysis to identify the optimal number of trisomies, and best subset regression and multivariate analysis to identify the optimal combination. Survival analysis considered three endpoints, as follows: event-free survival, defined as time to relapse, second tumour, or death, censored at last contact; relapse rate, defined as time to relapse for those reaching complete remission, censored at death in remission or last contact; and overall survival, defined as time to death, censored at last contact.

**Findings:**

The median follow-up time for UKALL97/99 was 10·59 years (IQR 9·25–12·06) and 9·40 years (8·00–11·55) for UKALL2003. UKALL97/99 included 208 female patients and 248 male patients, and UKALL2003 included 345 female patients and 380 male patients. We deduced that the trisomic status of four chromosomes provided the optimal information for predicting outcome. The good risk profile comprised karyotypes with +17 and +18 or +17 or +18 in the absence of +5 and +20. All remaining cases were classified in the poor risk profile. The ratio of patients with good risk and poor risk was 82:18 and 80:20 in the discovery and validation cohorts, respectively. In the validation cohort, patients with the high hyperdiploid good risk profile had an improved response to treatment compared with other patients with high hyperdiploidy at 10 years (relapse rate 5% [95% CI 3–7] *vs* 16% [10–23]; p<0·0001; event-free survival 92% [90–94] *vs* 81% [73–86]; p<0·0001; and overall survival 96% [94–97] *vs* 86% [79–91]; p<0·0001). The outcome for high hyperdiploid poor risk patients was similar to that of patients with an intermediate cytogenetic profile. The prognostic effect of the UKALL high hyperdiploid profile was independent of minimal residual disease and the profile outperformed other high hyperdiploid risk profiles.

**Interpretation:**

Future clinical trials and treatment protocols using high hyperdiploidy as a risk stratification factor should consider modifying the definition beyond chromosome count to incorporate this novel UKALL high hyperdiploid profile.

**Funding:**

Blood Cancer UK.

## Introduction

High hyperdiploidy is the most common cytogenetic abnormality in childhood acute lymphoblastic leukaemia, and accounts for 30–35% of B-cell precursor acute lymphoblastic leukaemia cases.^1^ High hyperdiploidy is defined by the non-random gain of chromosomes, increasing the modal chromosome number of leukaemic blasts from 46 to between 51 and 65 or 67.^2^, ^3^, ^4^ A DNA index of more than 1·16 is also frequently used to defined this subgroup. Gain of chromosomes X, 4, 6, 10, 14, 17, 18, and 21 accounts for more than 75% of aneuploidy events.^5^ High hyperdiploidy has been associated with a favourable outcome (survival >90%) in multiple studies over several decades.^1^, ^6^, ^7^, ^8^ Despite this association with good outcomes, the high frequency of patients with high hyperdiploid acute lymphoblastic leukaemia means that this subgroup accounts for up to 25% of all relapses.^9^ Therefore, identification of robust risk factors within this group is clinically relevant.

**Research in context**

**Evidence before this study**

Among children with acute lymphoblastic leukaemia, high hyperdiploidy—defined by a modal chromosome number of 51–65 chromosomes—is the largest subtype. Despite a strong association with good outcomes, the size of this subgroup means that a large number of patients relapse in absolute terms. Many studies have sought to define a smaller subgroup of these patients that is associated with a uniform very low risk of relapse and can be considered for treatment reduction. However, robust validation studies are rare and there is no consensus definition of low-risk high hyperdiploidy. Before this study, we searched PubMed for all publications citing high hyperdiploidy before March 30, 2020, using the search terms “high hyperdiploidy”, “acute lymphoblastic leukaemia”, “childhood”, “prognosis”, and “HeH”. This search was updated on January 10, 2021. All abstracts were screened by AE and reviewed in detail by AE and AVM.

**Added value of this study**

To our knowledge, this is the most comprehensive analysis to date of risk factors in childhood high hyperdiploid acute lymphoblastic leukaemia. We identified and validated a clinically useful profile that redefines low-risk high hyperdiploid acute lymphoblastic leukaemia. Our profile identified that most of the high-risk relapses in this subgroup of patients were based on four constituent trisomies: +5, +17, +18, +20. These trisomies have previously been proposed as risk factors in patients with high hyperdiploid acute lymphoblastic leukaemia. This novel profile outperformed previously published risk profiles in terms of prediction accuracy and prognostic impact, and although statistically independent of minimal residual disease, it could be further refined by its integration. The proposed profile identified low-risk patients with high hyperdiploid acute lymphoblastic leukaemia who should be considered for treatment de-intensification and a group of patients with high hyperdiploid acute lymphoblastic leukaemia who should be treated with other intermediate risk patients.

**Implications of all the available evidence**

The outcomes for patients with high hyperdiploidy are heterogeneous. Simply counting the number of chromosomes does not identify a subset of patients with a uniform outcome. There is evidence from this study and others that the pattern of chromosome gain can be used to define subgroups of patients with high hyperdiploidy with a distinct risk of relapse. The UKALL-high hyperdiploidy profile is simple to compute from a full karyotype or single nucleotide polymorphism array profile and outperforms existing profiles with high hyperdiploidy. We propose that this new definition be used prospectively to define patients with high hyperdiploid acute lymphoblastic leukaemia who have an excellent chance of a potential cure.

Numerous studies have examined cytogenetic risk factors within the subgroup of patients with high hyperdiploid acute lymphoblastic leukaemia, ranging from modal chromosome number to specific trisomies and the presence of structural abnormalities.^1^, ^6^, ^10^, ^11^, ^12^, ^13^, ^14^, ^15^, ^16^ Studies have shown improved outcomes for patients with a higher modal chromosome number^10^, ^11^, ^12^ and specific trisomies (+6;^13^ +4, +10;^14^ +10, +17;^6^ +18^1^, ^15^) and triple trisomies (+4, +10, +17),^16^ which often are related to each other.^17^ However, none of these studies investigated the pattern of all possible combinations of chromosomal gains and there is no consensus regarding the optimal risk factors in patients with high hyperdiploid acute lymphoblastic leukaemia. Additionally, the aforementioned risk factors have not been assessed within the context of end of induction minimal residual disease (MRD) risk stratification,^18^ which is now commonplace in acute lymphoblastic leukaemia treatment protocols.

In this study, we evaluated all previously published high hyperdiploid acute lymphoblastic leukaemia data from the UKALL97/99 and UKALL2003 studies and did a comprehensive analysis to identify new risk profiles by investigating all possible combinations of gained chromosomes. Additionally, we defined and validated a novel profile to be relevant in the context of risk stratification using MRD.

## Methods

### Study design and participants

In this retrospective analysis, patients were diagnosed with B-cell precursor acute lymphoblastic leukaemia by standard flow-cytometric criteria and were treated in the UKALL97/99 (between Jan 1, 1997, and June 15, 2002) or UKALL2003 (between Oct 1, 2003, and June 30, 2001) treatment trials, as previously described (appendix pp 22–23). All patients aged 1–18 years (in UKALL97/99) and 1–24 years (in UKALL2003) from the UK and Ireland who were newly diagnosed with B-cell precursor acute lymphoblastic leukaemia were eligible for the trials. In UKALL2003, MRD was evaluated by real-time qPCR analysis of immunoglobulin and T-cell receptor gene rearrangements).^18^ Cytogenetics and fluorescence in-situ hybridisation were done on pre-treatment bone marrow samples by the member laboratories of the UK Cancer Cytogenetics Group or centrally by the Leukaemia Research Cytogenetics Group, and results were reported with established nomenclature and definitions.^1^ Each trial was approved by the relevant ethics committee and patients or parents or guardians gave written informed consent in accordance with the Declaration of Helsinki.

### Procedures

Patients with high hyperdiploidy and concomitant *BCR–ABL1, ETV6–RUNX1, KMT2A*, or *TCF3–PBX1* fusions were excluded from this analysis on the assumption that the fusion gene was the primary genetic abnormality and would be used to direct therapy. All karyotypes were scrutinised for cases of masked hypodiploidy and, if found, were removed from the cohort.^19^

To develop the UKALL high hyperdiploidy prognostic profile, we used the two trial datasets as discovery (UKALL97/99) and validation (UKALL2003) cohorts. To ensure the development of a robust profile, only chromosomal abnormalities and trisomies visible by conventional G-banded karyotyping were included in the analyses. To simulate the real-world setting, we validated the profile using cytogenetic data generated as part of the standard-of-care genetic tests done by regional genetic laboratories for UKALL2003 patients. The SALSA MLPA kit P335 (MRC Holland; Amsterdam, Netherlands), which includes probes directed to *IKZF1*, *CDKN2A/B*, *PAX5*, *EBF1*, *ETV6*, *BTG1*, *RB1*, and *PAR1* (*P2RY8*/*CSF2RA*/*IL3RA*/*CRLF2*) was used to identify copy number alterations.

### Statistical analysis

Initially, the evaluation of previously reported risk factors in high hyperdiploidy was done using the UKALL2003 trial only, because this trial had used MRD to direct therapy. The development of the UKALL high hyperdiploid profile was done using discovery (UKALL97/99) and validation (UKALL2003) cohorts.

We examined modal chromosome number both as a continuous variable and across three predefined categories (51–53, 54–57, and 58–65).^11^, ^12^ We also examined the prognostic impact of double trisomies (+4, +10) and triple trisomies (+4, +10, +17) as proposed by the Children's Oncology Group (COG).^6^, ^16^ Previously, Heerema and colleagues^17^ proposed subgroups of high hyperdiploidy based on the pattern of chromosomal gains and modal chromosome number. We replicated this approach using correlation coefficients to identify clusters of gained chromosomes and their correlation with relapse. We analysed age, white cell count, and MRD as both continuous and categorical variables. Survival analysis considered three endpoints, as follows: event-free survival, defined as time to relapse, second tumour, or death, censored at last contact; relapse rate, defined as time to relapse for those reaching complete remission, censored at death in remission or last contact; and overall survival, defined as time to death, censored at last contact. The median follow-up times for the discovery and validation cohorts were 10·59 years (IQR 9·25–12·06) and 9·40 years (8·00–11·55), respectively. We calculated and compared survival rates at 10 years (unless otherwise specified) using Kaplan-Meier methods, log-rank tests, and Cox regression models (univariate and multivariate analyses). p<0·05 was considered to indicate a significant difference. We compared the outcomes of patients with and without a given chromosomal gain, using a univariate Cox regression model.

To investigate the optimal number of chromosomal gains, we used area under the receiver operating characteristic curve (AUC), generalised linear models,^20^ and targeted projection pursuit.^21^ We used best subset regression to evaluate the best subset models.^20^ We measured Mallows’ C_p_ for each combination and the model with the lowest value was chosen as the fittest. To assess the optimal combination of chromosomes for predicting outcome, multivariate analysis, generalised linear models, network analysis, and coefficient of the risk model were used. The model with the smallest Bayesian information criterion with a forward stepwise criteria score over the complete set of possible models was deemed the least complex and fittest model (appendix p 24).^22^ We plotted the risk of relapse against unique MRD thresholds for all patients. Additionally, cumulative distribution function for each group was plotted to identify the optimal discriminative threshold. Hazard ratios [HRs] comparing relapse rate, event-free survival, and overall survival between subgroups were calculated using univariate and multivariate Cox models. Finally, we did a sensitivity analysis to confirm the utility of the profile in key patient subgroups.

We did all survival analyses using Stata 14.0 and we did all subsequent analyses using R 3.4.4 (appendix p 24).

### Role of the funding source

The funder of the study had no role in study design, data collection, data analysis, data interpretation, or writing of the report.

## Results

**Figure 1:**
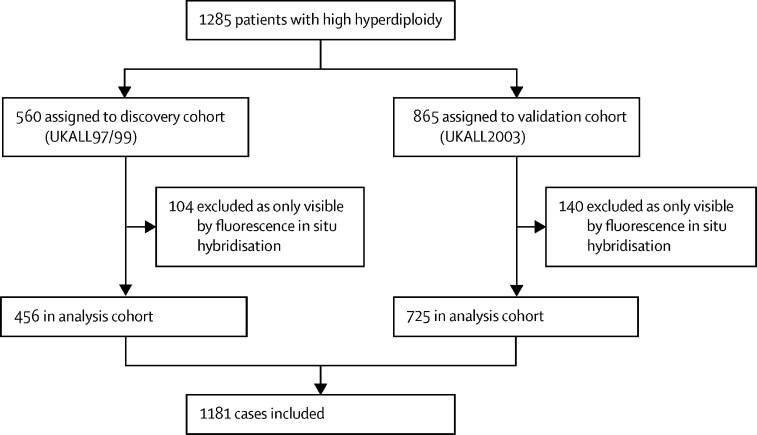
Discovery and validation of novel high hyperdiploid acute lymphoblastic leukaemia subgroups

Data from 456 patients from the UKALL97/99 cohort and 725 patients from the UKALL2003 cohort were analysed (figure 1) and a summary of the patient characteristics of both cohorts is shown in table 1. Using the UKALL2003 cohort, we evaluated the prognostic effect of previously published cytogenetic risk factors. We did not observe any significant correlation with any of the three survival endpoints examined and the modal chromosome number (appendix pp 20–21). Six trisomies—+3, +5, +7, +17, +18 and +20—were associated with outcomes (appendix p 10). Trisomies of 5 and 18 were significant for all three endpoints, whereas trisomies of 7, 17, and 20 were significant for only one endpoint each, event-free survival, relapse rate, and overall survival, respectively. Among UKALL2003 patients, double trisomies and triple trisomies were associated with a significantly better relapse rate but not event-free survival and overall survival compared with other patients with high hyperdiploidy (HRs for relapse rate 0·51 [95% CI 0·28–0·91]; p=0·024 and 0·38 [0·19–0·77]; p=0·0070 for double trisomies and triple trisomies, respectively; appendix p 20). Like Heerema and colleagues,^17^ we identified five distinct clusters of chromosomes (appendix p 13), which mapped closely to the five groups reported by Heerema and colleagues,^17^ supporting the notion that chromosomes are gained non-randomly and within groups. Moreover, we showed that the risk of relapse varied by cluster (appendix p 21). Specifically, group II (HR 3·16 [95% CI 1·32–7·54]; p=0·0093) and group V (3·64 [1·46–9·05]; p=0·0054), which comprised 130 (18%) and 91 (13%) cases, respectively, showed a significant increase in relapse rate compared with group I.

**Table 1:** Demographics and outcomes of patients with high hyperdiploid acute lymphoblastic leukaemia treated in UKALL97/99 and UKALL2003

		**UKALL97/99 cohort**				**UKALL2003 cohort**			
		Total (n=456)	UKALL-HeH good risk (n=373)	UKALL-HeH poor risk (n=83)	p value*	Total (n=725)	UKALL-HeH good risk (n=579)	UKALL-HeH poor risk (n=146)	p value*
Sex		..	..	..	0·60	..	..	..	0·64
	Female	208 (46%)	168 (45%)	40 (48%)	..	345 (48%)	278 (48%)	67 (46%)	..
	Male	248 (54%)	205 (55%)	43 (52%)	..	380 (52%)	301 (52%)	79 (54%)	..
Age, years		..	..	..	0·62	..	..	..	0·19
	1–9	409 (90%)	337 (90%)	72 (87%)	..	614 (85%)	497 (86%)	117 (80%)	..
	10–14	38 (8%)	29 (8%)	9 (11%)	..	72 (10%)	52 (9%)	20 (14%)	..
	≥15	9 (2%)	7 (2%)	2 (2%)	..	39 (5%)	30 (5%)	9 (6%)	..
US National Cancer Institute risk group		..	..	..	0·57	..	..	..	0·42
	Standard	362 (79%)	298 (80%)	64 (77%)	..	550 (76%)	443 (77%)	107 (73%)	..
	High	94 (21%)	75 (20%)	19 (23%)	..	175 (24%)	136 (23%)	39 (27%)	..
White cell count, 10^9^ per L		..	..	..	0·54	..	..	..	0·35
	0–49	407 (89%)	332 (89%)	75 (90%)	..	656 (90%)	523 (90%)	133 (91%)	..
	50–99	35 (8%)	28 (8%)	7 (8%)	..	53 (7%)	45 (8%)	8 (5%)	..
	≥100	14 (3%)	13 (3%)	1 (1%)	..	16 (2%)	11 (2%)	5 (3%)	..
Modal chromosome number		..	..	..	<0·0001	..	..	..	<0·0001
	Median (IQR)	55 (54–57)	55 (54–57)	53 (52–56)	..	55 (54–56)	55 (54–56)	53 (52–56)	..
	51–53	87 (19%)	50 (13%)	37 (45%)	..	120 (17%)	74 (13%)	46 (32%)	..
	54–57	226 (50%)	199 (53%)	27 (33%)	..	383 (53%)	347 (60%)	36 (25%)	..
	58–65	84 (18%)	72 (19%)	12 (14%)	..	92 (13%)	71 (12%)	21 (14%)	..
	Unclassifiable†	59 (13%)	52 (14%)	7 (8%)	..	130 (18%)	87 (15%)	43 (29%)	..
Copy number alteration risk‡		..	..	..	0·040	..	..	..	0·29
	Good	175/225 (78%)	150/187 (80%)	25/38 (66%)	..	182/239 (76%)	147/189 (78%)	35/50 (70%)	..
	Intermediate	38/225 (17%)	30/187 (16%)	8/38 (21%)	..	40/239 (17%)	31/189 (16%)	9/50 (18%)	..
	Poor	12/225 (5%)	7/187 (4%)	5/38 (13%)	..	17/239 (7%)	11/189 (6%)	6/50 (12%)	..
Risk profile									
	Double trisomy (+4, +10)	251 (55%)	220 (59%)	31 (37%)	<0·0001	395 (54%)	350 (60%)	45 (31%)	<0·0001
	Triple trisomy (+4, +10, +17)	195 (43%)	185 (50%)	10 (12%)	<0·0001	299 (41%)	291 (50%)	8 (5%)	<0·0001
Relapse rate									
	Rate at 10 years (95% CI)	13% (11–17)	12% (9–15)	28% (20–39)	<0·0001	7% (5–9)	5% (3–7)	16% (10–23)	<0·0001
	Patients relapsed	67 (15%)	44 (12%)	23 (28%)	..	47 (6%)	25 (4%)	22 (15%)	..
	HR (95% CI)	2·50 (1·51–4·14)	..	..	<0·0001§	3·80 (2·14–6·75)	..	..	<0·0001§
Event-free survival									
	Rate at 10 years (95% CI)	84% (81–87)	86% (82–89)	71% (60–79)	0·0030	90% (87–92)	92% (90–94)	81% (73–86)	<0·0001
	Events in patients	80 (18%)	56 (15%)	24 (29%)	..	72 (10%)	44 (8%)	28 (19%)	
	HR (95% CI)	2·04 (1·27–3·30)	..	..	0·0030§	2·68 (1·67–4·30)	..	..	<0·0001§
Overall survival									
	Rate at 10 years (95% CI)	93% (91–95)	94% (91–96)	89% (80–94)	0·070	94% (92–95)	96% (94–97)	86% (79–91)	<0·0001
	Patients died	42 (9%)	30 (8%)	12 (14%)	..	42 (6%)	23 (4%)	19 (13%)	..
	HR (95% CI)	1·82 (0·93–3·55)	..	..	0·080§	3·42 (1·86–6·27)	..	..	<0·0001§

Data are n (%) or n/N (%), unless otherwise indicated. HeH=high hyperdiploidy. HR=hazard ratio.

* Most of these p values are from Fisher's exact test.

† These karyotypes had a modal chromosome range which prevented accurate classification.

‡ As defined by Hamadeh and colleagues;^23^ denominators represent the 225 tested cases in the discovery cohort and 239 tested cases in the validation cohort.

§ p values from log-rank test for equality of the survival functions.

We examined the effect of age, white cell count, and MRD as both continuous and categorical variables. Among patients with high hyperdiploid acute lymphoblastic leukaemia treated in UKALL2003, neither white cell count (HR 1·22 [95% CI 0·95–1·55]; p=0·12) nor age (1·04 [0·98–1·11]; p=0·19) were correlated with relapse rate (appendix p 20). Increasing age (HR 1·08 [95% CI 1·01–1·14]; p=0·020), but not white cell count (1·03 [0·79–1·34]; p=0·81), was associated with an increased risk of death (appendix p 20). Patients with high hyperdiploid acute lymphoblastic leukaemia with end of induction MRD of 0·01% or greater had inferior relapse rates (HR 2·26 [95% CI 1·25–4·08]; p=0·0070), event-free survival (2·30 [1·11–4·75]; p=0·020), and survival rates (3·48 [1·44–8·39]; p=0·0051; appendix p 21) compared with patients who had MRD less than 0·01%. We previously reported that the predictive value of MRD was optimal when considered as a continuous variable.^18^ Among the UKALL2003 cohort, each log reduction of end of induction MRD was associated with a reduced risk of relapse (HR 0·85 [95% CI 0·77–0·93]; p<0·0001). To further extend the analysis of MRD as a continuous variable, we examined the distribution of MRD for patients with specific trisomies (appendix p 12). Patients with trisomies X, 9, 10, 11, 12, 18, and 22 had MRD distributions that were distinct from the whole high hyperdiploid group (9 and X higher MRD, and 10, 11, 12, 18, and 22 lower MRD).

Next, we sought to identify the optimal number of chromosomes required to maximise prediction. Using the discovery cohort (UKALL97/99), we generated all possible combinations of up to six gained chromosomes and compared them using the C-index as a measure of the prediction power of the profile. The prediction performance of the models increased with each additional chromosome up to four chromosomes, after which no additional benefit was gained from adding more chromosomes (appendix p 5). We found further support for this result using Mallows C_p_ statistic (appendix p 14).

We explored which combination of four chromosomes provided the optimal information for predicting outcome. Using the discovery cohort, we used univariate Cox models to identify five chromosomes (5, 11, 17, 18, and 20) as being the most informative (appendix p 6). We used multivariate stepwise Cox regression modelling to produce a final model that comprised four chromosomes—5, 17, 18, and 20. We validated this result using the Bayesian information criterion (appendix p 15). Correlation network analysis (appendix p 16) and volcano plot (appendix p 17) supported that these chromosomes had the greatest prognostic impact.

**Figure 2:**
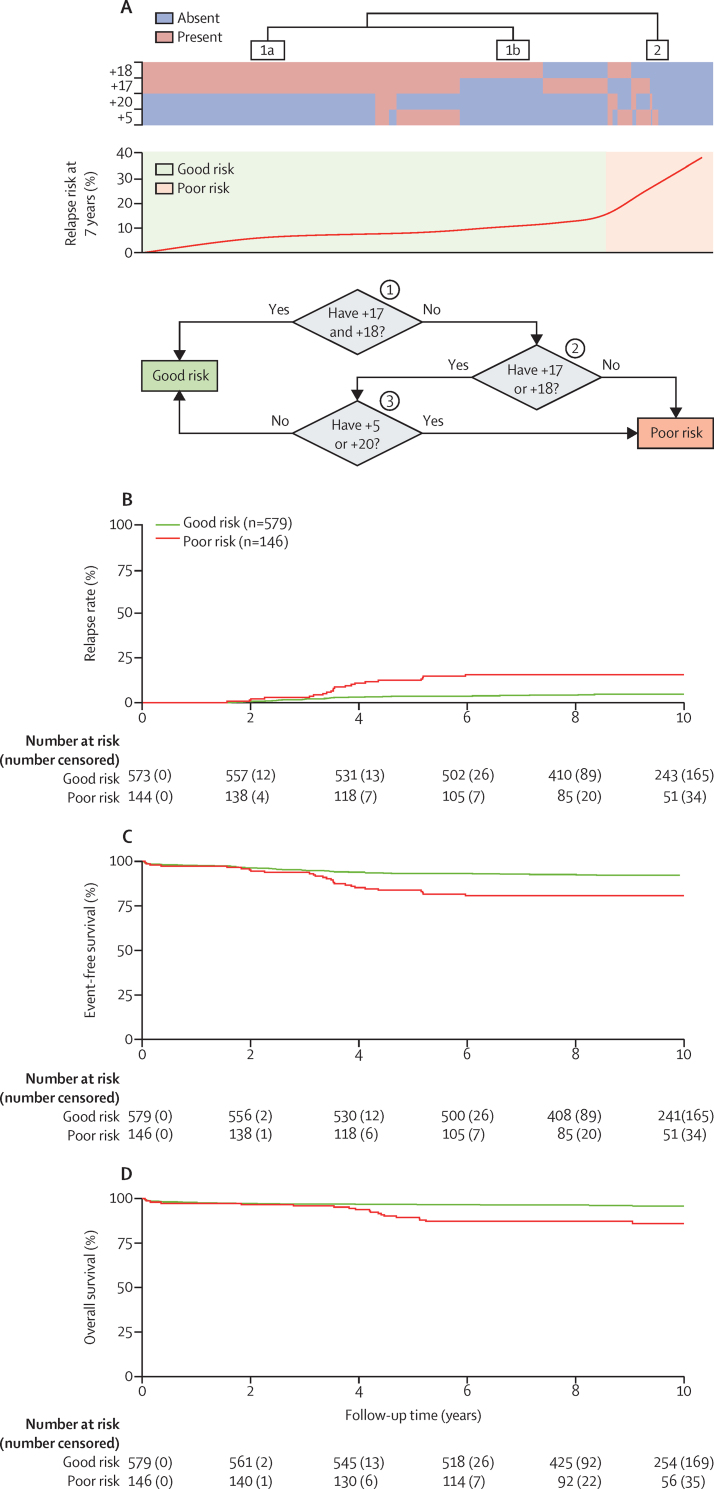
Overview of the novel high hyperdiploid risk profile (A) The top panel shows the correlation between relapse rate and cases with trisomy 5, 17, 18, and 20 for patients in the discovery cohort (UKALL97/99). There are two distinct clusters: 1 and 2. Based on the pattern of the gains and corresponding relapse rate a decision tree (bottom panel) was constructed. Kaplan-Meier survival curves for patients in the validation cohort (UKALL2003) for event-free survival (B), relapse rate (C), and overall survival (D).

Therefore, the optimal set of chromosomes for predicting relapse was 5, 17, 18, and 20. The good risk profile comprised patients with both +17 and +18 together or patients with either +17 or +18 coupled with an absence of +5 or +20 (figure 2A). The remaining patients were classified as having a poor risk profile and were characterised either by the absence of +17 or +18 or the presence of just one in combination with +5 or +20. Overall, the high hyperdiploid good risk cluster comprised 373 (82%) of 456 patients in the discovery cohort whereas the high hyperdiploid poor risk cluster comprised 83 (18%) of 456 patients. We found no correlation between high hyperdiploid risk group (good risk *vs* poor risk) and age, sex, or white cell count (table 1). In the discovery cohort, patients with a high hyperdiploid poor risk profile had a significant increase in relapse rate (HR 2·50 [95% CI 1·51–4·14]; p<0·0001) and a significant decrease in event-free survival (2·04 [1·27–3·30]; p=0·0030), but no difference in overall survival (1·82 [0·93–3·55]; p=0·080; table 1) compared with those with a good risk profile.

We validated this risk profile using patients treated in the UKALL2003 trial. The relative size of the high hyperdiploid good risk and high hyperdiploid poor risk groups were similar in the validation and discovery cohorts (82% *vs* 80% and 18% *vs* 20% in UKALL97/99 and UKALL2003, respectively; table 1). As in the discovery cohort, we found no correlation between high hyperdiploid risk group (good risk *vs* poor risk) and age, sex, or white cell count in the validation cohort (table 1). Patients with a high hyperdiploid poor risk profile had an increased risk of relapse (HR 3·80 [95% CI 2·14–6·75]; p<0·0001), events (2·68 [1·67–4·30]; p<0·0001), and death (3·42 [1·86–6·27]; p<0·0001) compared with patients in the high hyperdiploid good risk group (table 1; figure 2B–D).

MRD is one of the key risk factors used to direct therapy in patients with childhood acute lymphoblastic leukaemia. Therefore, we examined the interaction of MRD and the risk profile in UKALL2003. The distribution of patients across different MRD categories was similar between the two high hyperdiploidy risk groups despite the difference in outcome (table 2). However, for most of the MRD categories examined, including the 0·01% threshold used to stratify patients in the trial, patients in the high hyperdiploid poor risk group had an inferior outcome (table 2). We previously showed that the optimal prognostic MRD threshold varies by genetic risk group.^18^ Threshold analysis^22^ revealed MRD of 0·03% to be the optimal threshold for patients with high hyperdiploid acute lymphoblastic leukaemia in UKALL2003 (appendix p 18). Stratifying patients by this MRD threshold and the UKALL high hyperdiploid profile revealed that both risk factors were highly informative for predicting relapse (table 2). Importantly, patients with MRD less than 0·03% who were also in the UKALL high hyperdiploid good risk profile group accounted for 377 (60%) of 632 patients with high hyperdiploidy, with a relapse rate of 4% (95% CI 2–6), an event-free survival rate of 95% (92–97), and an overall survival rate of 98% (96–99; table 2; appendix p 9).

**Table 2:** Outcome of patients with high hyperdiploid acute lymphoblastic leukaemia treated in UKALL2003 stratified by UKALL high hyperdiploid risk profile, end of induction minimal residual disease, and UKALL copy number alteration profile

			**Number of cases**			**Relapse rate**				**Event-free survival**				**Overall survival**			
			Total	UKALL-HeH good risk	UKALL-HeH poor risk	Total	UKALL-HeH good risk	UKALL-HeH poor risk	p value	Total	UKALL-HeH good risk	UKALL-HeH poor risk	p value	Total	UKALL-HeH good risk	UKALL-HeH poor risk	p value
All patients with high hyperdiploidy			725 (100%)	579 (100%)	146 (100%)	7% (5–9)	5% (3–7)	16% (10–23)	0·0030	90% (87–92)	92% (90–94)	81% (73–86)	0·0010	94% (92–95)	96% (94–97)	86% (79–91)	<0·0001
MRD data available			632 (87%)	510 (88%)	122 (84%)	7% (5–9)	5% (3–7)	16% (10–24)	<0·0001	91% (88–93)	93% (90–95)	81% (73–87)	<0·0001	95% (93–96)	97% (95–98)	86% (78–92)	<0·0001
MRD data not available			93 (13%)	69 (12%)	24 (16%)	6% (3–15)	4% (1–13)	14% (5–37)	0·079	84% (74–90)	85% (74–92)	79% (57–91)	0·53	87% (78–92)	88% (78–94)	83% (61–93)	0·59
MRD levels, %																	
	0		174 (27%)	145 (28%)	29 (24%)	4% (2–9)	3% (1–9)	7% (2–26)	0·19	95% (91–98)	97% (91–99)	89% (71–96)	0·037	98% (95–99)	100%	89% (71–96)	<0·0001
	0 to <0·01		196 (31%)	158 (31%)	38 (31%)	6% (3–10)	4% (2–9)	15% (6–32)	0·0030	92% (87–95)	94% (89–97)	81% (63–90)	0·0020	95% (91–98)	98% (94–99)	85% (66–93)	0·0020
	0·01 to <0·1		154 (24%)	126 (25%)	28 (23%)	7% (4–12)	3% (1–9)	23% (11–44)	<0·0001	91% (86–95)	94% (89–97)	77% (56–89)	0·0070	95% (89–98)	95% (89–98)	92% (73–98)	0·45
	0·1 to <1·0		73 (12%)	54 (11%)	19 (16%)	11% (6–22)	12% (5–24)	11% (3–38)	0·92	84% (73–90)	83% (70–91)	84% (59–95)	0·91	93% (84–97)	94% (84–98)	89% (64–97)	0·45
	≥1·0		35 (6%)	27 (5%)	8 (7%)	19% (9–38)	12% (4–34)	42% (16–82)	0·048	76% (58–87)	81% (60–92)	58% (18–84)	0·20	79% (61–89)	85% (65–94)	57% (17–84)	0·10
Original MRD risk group, %																	
	<0·01		370 (59%)	303 (59%)	67 (55%)	5% (3–8)	4% (2–7)	11% (6–23)	0·0010	93% (90–96)	95% (92–97)	84% (73–91)	<0·00001	97% (94–98)	99% (97–100)	86% (73–93)	<0·0001
	≥0·01		262 (41%)	207 (41%)	55 (45%)	10% (7–14)	7% (4–11)	21% (12–35)	0·0020	87% (82–91)	90% (85–93)	77% (63–86)	0·023	92% (88–95)	94% (89–96)	87% (74–93)	0·076
Optimal MRD threshold, %																	
	<0·03		462 (73%)	377 (74%)	85 (70%)	5% (3–8)	4% (2–6)	11% (6–21)	0·0010	93% (90–95)	95% (92–97)	85% (76–91)	<0·0001	96% (94–98)	98% (96–99)	88% (77–94)	<0·0001
	≥0·03		170 (27%)	133 (26%)	37 (30%)	12% (8–18)	8% (4–14)	27% (15–45)	0·0030	84% (78–89)	88% (81–92)	71% (53–84)	0·024	90% (85–94)	92% (86–96)	83% (65–92)	0·064
Slow early responder*																	
	Yes		68 (9%)	53 (9%)	15 (10%)	19% (11–32)	12% (5–26)	46% (24–75)	0·0010	76% (63–85)	83% (69–91)	50% (23–72)	0·0040	86% (76–93)	94% (83–98)	57% (29–78)	<0·0001
	No		657 (91%)	526 (91%)	131 (90%)	6% (4–8)	4% (3–6)	12% (8–20)	<0·0001	91% (89–93)	93% (90–95)	84% (77–90)	0·0010	95% (92–96)	96% (94–97)	89% (82–94)	0·0040
Treatment pathways†																	
	MRD <0·01% and regimen A or B		346	287	59	5% (3–8)	4% (2–7)	9% (4–21)	0·014	94% (90–96)	95% (92–97)	86% (74–93)	0·0010	97% (94–98)	99% (96–100)	88% (74–95)	<0·0001
		One delayed intensification block	158 (46%)	133 (46%)	25 (42%)	95% (89–98)	95% (89–98)	92% (71–98)	0·31	5% (2–10)	5% (2–11)	4% (1–27)	0·90	97% (90–99)	99% (92–100)	85% (48–97)	0·011
		Two delayed intensification blocks	188 (54%)	154 (54%)	34 (57%)	92% (88–95)	95% (90–97)	82% (64–91)	0·0020	5% (3–9)	3% (1–8)	13% (5–30)	0·0040	97% (93–99)	99% (95–100)	88% (71–95)	0·0020
	MRD ≥0·01% and regimen A or B		143	113	30	8% (5–14)	6% (3–13)	15% (6–35)	0·17	90% (84–94)	92% (85–96)	82% (62–92)	0·14	94% (88–97)	94% (87–97)	93% (75–98)	0·69
	MRD ≥0·01% and regimen C		151	116	35	11% (7–18)	7% (3–14)	27% (15–46)	0·0010	84% (77–89)	88% (80–92)	71% (52–83)	0·021	90% (84–94)	94% (88–97)	76% (58–87)	0·0040
UKALL copy number alteration profile‡																	
	Total		239 (100%)	189 (100%)	50 (100%)	10% (7–15)	6% (3–11)	27% (16–42)	<0·0001	86% (81–90)	90% (85–94)	69% (53–80)	<0·0001	92% (87–95)	94% (90–97)	81% (66–90)	0·0020
	Good risk		182 (76%)	147 (78%)	35 (70%)	9% (5–14)	5% (2–10)	25% (13–44)	<0·0001	88% (82–92)	92% (87–96)	68% (50–81)	<0·0001	94% (89–96)	96% (91–98)	83% (65–92)	0·0020
	Intermediate or poor risk		57 (24%)	42 (22%)	15 (30%)	15% (8–28)	10% (4–25)	29% (12–61)	0·090	80% (67–89)	83% (68–92)	71% (39–88)	0·37	85% (73–92)	88% (74–95)	75% (41–91)	0·40

Data are n (%), rates at 10 years (95% CI), or n, unless otherwise indicated. HeH=high hyperdiploid. MRD=minimal residual disease. NCI=US National Cancer Institute.

* Slow early responders were patients who had ≥25% marrow blasts at day 15 (NCI standard risk) or day 8 (NCI high risk).

† Most patients followed one of these three main treatment pathways; the remaining patients have been excluded from this section for clarity. Details of treatment pathways can be found in the appendix (p 22).

‡ Only 239 patients were tested by multiplex ligation-dependent probe amplification to determine the UKALL copy number alteration profile.

Patients classified as slow early responders were treated on the high-risk protocol (regimen C) in UKALL2003. Slow early responders had a poorer outcome in terms of relapse rate compared with rapid early responders (19% [95% CI 11–32] *vs* 6% [4–8]; p=0·0003). Slow early responders with a high hyperdiploid good risk profile had a significantly lower relapse rate than did slow early responders with a high hyperdiploid poor risk profile (12% [95% CI 5–26] *vs* 46% [24–75]; p=0·0010; table 2). In UKALL2003, patients with MRD less than 0·01% were treated on the standard regimen (A or B) and received one or two delayed intensification blocks. The excellent outcome of patients with a high hyperdiploid good risk profile was maintained even when they were treated with the least intensive therapy (table 2). Patients with MRD of 0·01% were randomly assigned between staying on regimen A or B and regimen C or, if they had other risk factors, were assigned regimen C. The UKALL high hyperdiploid profile retained its prognostic effect among patients treated on these different pathways. The relapse rate for patients with a high hyperdiploid good risk profile with MRD of 0·01% or greater was similar whether patients received augmented (regimen C) or standard therapy (regimen A or B; table 2).The presence of some copy number alteration profiles can affect the outcome of patients with high hyperdiploid acute lymphoblastic leukaemia.^23^ Analysis of 239 patients with high hyperdiploid acute lymphoblastic leukaemia showed that most patients (182 [76%]) also had a good risk UKALL copy number alteration profile. Among these 182 patients, those with a UKALL high hyperdiploid poor risk profile had a higher relapse rate compared with patients with a UKALL high hyperdiploid good risk profile (5%, 95% CI 2–10 *vs* 25%, 13–44; p<0·0001; table 2). Among the tested cases we identified 18 *IKZF1* deletions, 13 in the high hyperdiploid good risk group and five in the high hyperdiploid poor risk group. Sensitivity analysis revealed the UKALL high hyperdiploid profile (the profile derived from both the discovery and validation cohorts) to be prognostic in all relevant patient subgroups and across the three main treatment groups (appendix p 19).

To determine the best risk-based definition of high hyperdiploidy, we compared the UKALL high hyperdiploid good risk group with the triple trisomy and double trisomy groups used by the COG in the USA (table 3). The UKALL high hyperdiploid good risk profile identified a cohort of patients with high hyperdiploidy with similar demographics, responses, and outcomes, but which accounted for a much higher proportion of the entire high hyperdiploid population as defined by modal chromosome number (table 3). As the UKALL high hyperdiploid good risk profile was considerably larger than the proportions of patients with triple trisomy and double trisomy, it captured a higher proportion of relapses (table 3). However, most of these relapses were standard risk and hence had a high chance of salvage.^9^ 47 relapses occurred in the UKALL2003 high hyperdyploid cohort and five (11%) were classified as high risk.^9^ Among all patients with high hyperdiploidy, Cox regression analysis adjusted and unadjusted for MRD confirmed that the UKALL high hyperdiploid good risk profile was more discriminatory than both triple trisomy and double trisomy for predicting outcome (table 3). Both C-index and AUC measurements were higher for the UKALL high hyperdiploid profile than for triple trisomy and double trisomy (table 3; appendix pp 7–8).

**Table 3:** Comparison of the novel UKALL high hyperdiploid group with double trisomy and triple trisomy definitions proposed by the Children's Oncology Group

		**UKALL HeH good risk, n=579**	**UKALL HeH good risk and MRD <0·03%, n=377**	Triple trisomy (+4,+10,+17), n=299*	Double trisomy (+4,+10), n=395*
Proportion of high hyperdiploidy cases†		80%	60%	41%	54%
Sex					
	Female	278 (48%)	179 (47%)	147 (49%)	198 (50%)
	Male	301 (52%)	198 (53%)	152 (51%)	197 (50%)
US National Cancer Institute risk group					
	Standard	443 (77%)	281 (75%)	229 (77%)	298 (75%)
	High	136 (23%)	96 (25%)	70 (23%)	97 (25%)
Slow early responders					
	No	526 (91%)	350 (93%)	272 (91%)	357 (90%)
	Yes	53 (9%)	27 (7%)	27 (9%)	38 (10%)
MRD at end of induction, %					
	<0·03	377 (65%)	377 (100%)	187/255 (73%)	247/337 (73%)
	≥0·03	202 (35%)	..	68/255 (27%)	90/337 (27%)
Relapse					
	No	548 (96%)	364 (97%)	284/294 (97%)	372 (95%)
	Yes	25 (4%)	13 (3%)	10/294 (3%)	18 (5%)
	Proportion of all HeH relapses	53%	28%	21%	38%
Outcome rates at 10 years (95% CI)					
	Relapse rate	5% (3–7)	4% (2–6)	4% (2–7)	5% (3–8)
	Event-free survival	92% (90–94)	95% (92–97)	92% (88–94)	91% (88–94)
	Overall survival	96% (94–97)	98% (96–99)	96% (93–97)	95% (92–96)
Unadjusted HR comparing each group with remaining high hyperdiploid cases					
	Relapse rate	0·26 (0·15–0·47); <0·0001	0·28 (0·14–0·53); <0·0001	0·38 (0·19–0·77); 0·0070	0·51 (0·28–0·91); 0·024
	Event-free survival	0·37 (0·23–0·60); <0·0001	0·29 (0·17–0·51); <0·0001	0·71 (0·44–1·16); 0·176	0·74 (0·47–1·18); 0·203
	Overall survival	0·29 (0·16–0·54); <0·0001	0·16 (0·07–0·39), <0·0001	0·64 (0·33–1·24); 0·185	0·84 (0·46–1·53); 0·56
HR comparing each group with remaining high hyperdiploid cases adjusted for MRD‡					
	Relapse rate	0·26 (0·14–0·48); <0·0001	0·31 (0·16–0·60); <0·0001	0·41 (0·20–0·86); 0·018	0·54 (0·29–1·02); 0·057
	Event-free survival	0·33 (0·20–0·56); <0·0001	0·31 (0·18–0·55); <0·0001	0·64 (0·36–1·13); 0·123	0·69 (0·41–1·16); 0·164
	Overall survival	0·23 (0·11–0·48); <0·0001	0·18 (0·07–0·44); <0·0001	0·47 (0·20–1·11); 0·086	0·69 (0·34–1·44); 0·325
HR comparing each group with remaining high hyperdiploid cases for MRD at end of induction <0·03%					
	Relapse rate	0·27 (0·12–0·61); 0·0020	..	0·50 (0·20–1·28); 0·149	0·53 (0·23–1·23); 0·142
	Event-free survival	0·29 (0·14–0·59); 0·0010	..	0·69 (0·32–1·46); 0·329	0·61 (0·30–1·24); 0·172
	Overall survival	0·14 (0·05–0·40); <0·0001	..	0·53 (0·17–1·66); 0·276	0·57 (0·20–1·60); 0·285
HR comparing each group with remaining high hyperdiploid cases for MRD at end of induction ≥0·03%					
	Relapse rate	0·28 (0·11–0·68); 0·0050	..	0·27 (0·08–0·92); 0·037	0·50 (0·20–1·26); 0·142
	Event-free survival	0·41 (0·19–0·91); 0·029	..	0·54 (0·23–1·28); 0·163	0·74 (0·34–1·61); 0·453
	Overall survival	0·39 (0·14–1·10); 0·075	..	0·37 (0·11–1·32); 0·127	0·78 (0·28–2·16); 0·639
Prediction accuracy					
	C-index	0·64	0·66	0·60	0·59
	Area under the curve	0·64	0·65	0·61	0·59

Data are n (%), n/N (%), or HR (95% CI); p value, unless otherwise indicated. MRD data were unavailable for 93 patients. HeH=high hyperdiploid. HR=hazard ratio. MRD=minimal residual disease.

* Derived from UKALL2003.

† Proportion of all cases with 51–65 chromosomes.

‡ MRD was included in the model as a covariate.

## Discussion

High hyperdiploidy is the most prevalent genetic subgroup of B-cell positive acute lymphoblastic leukaemia.^2^, ^4^, ^15^ Although associated with a good outcome, high hyperdiploid relapse remains a major clinical problem. Many clinical trials now use high hyperdiploidy either alone or in combination with MRD to assign patients to risk groups. The original definition of high hyperdiploidy was based on the number of chromosomes, using 51 chromosomes as the threshold.^24^ Currently, the optimal definition of the good risk high hyperdiploid group is unclear. Some clinical study groups have continued to count chromosomes, whereas others have opted to use DNA index or the trisomic status of specific chromosome combinations.^3^ Several study groups have examined the prognostic effects of various risks to accurately define a good risk high hyperdiploid group that can be used to assist patient management.^3^, ^12^, ^13^, ^14^, ^25^ However, none of these studies adopted a comprehensive approach to their analysis and many lacked validation cohorts. In this study, we used two independent clinical trial cohorts to assess previous high hyperdiploid risk factors comprehensively and used novel techniques to develop and validate a robust risk profile on the basis of the copy number status of four chromosomes: 5, 17, 18, and 20. The key finding from this study was identification of a robust and simple profile to define a cohort of patients with high hyperdiploid acute lymphoblastic leukaemia who have a low relapse rate and good overall survival.

Our study highlighted several unique features that strengthen the robustness and applicability of our findings. First, we evaluated all previously identified markers and profiles in a cohort of patients treated on a contemporary MRD-driven protocol, UKALL2003. Second, we used a methodical and comprehensive approach to assess both the number and identity of trisomies to be considered when developing prognostic models using discovery and validation cohorts. Finally, we evaluated the novel profile within the context of MRD and the UKALL copy number alteration profile. We showed that our high hyperdiploid profile has more predictive power than similar classifications.

We propose that clinical trials that seek to define a good risk subgroup based on high hyperdiploidy should use our profile rather than rely on counting chromosomes. We estimate that around 80% of patients with high hyperdiploid acute lymphoblastic leukaemia have the high hyperdiploid good risk profile; therefore, around 25% of all patients with B-cell positive acute lymphoblastic leukaemia will fall into this category. The UKALL high hyperdiploid good risk group was larger than both the triple trisomy and double trisomy groups and yet had a similar good outcome. The outcome for the UKALL high hyperdiploid good risk group was similar to that reported for patients with *ETV6-RUNX1*-positive acute lymphoblastic leukaemia treated on the same trial.^18^ By contrast, the outcome of the remaining patients with high hyperdiploidy was more akin to that reported for patients with intermediate risk cytogenetics.^18^ Using the high hyperdiploid good risk profile to define good risk high hyperdiploidy, rather than modal chromosome number, would define a group of patients whose predicted outcome was better than a group defined by model chromosome number.

Even though the UKALL high hyperdiploid poor risk group accounted for just 20% of patients with high hyperdiploidy, it captured nearly half of those who went on to have high-risk relapse. The outcome of patients with high-risk relapse is poor, so it is important that such patients avoid any treatment reduction during frontline therapy. Applying the UKALL high hyperdiploid profile prospectively could ensure that patients at greatest risk of a high-risk relapse are assigned to the intermediate risk treatment group and avoid treatment reduction interventions or randomisations.

We previously reported that end of induction MRD was log-normally distributed and that different genetic subtypes had different distributions with distinct optimal MRD thresholds.^18^ As a result, we identified that among patients with high hyperdiploidy, 0·03% MRD was the optimal threshold for patients with high hyperdiploidy rather than the 0·01% cutoff used to direct therapy. The outcome of UKALL high hyperdiploid poor risk patients did not vary much by MRD, especially given the relatively modest number of patients in each category, and event-free survival rates were always less than 80%. However, two small overlapping very high-risk groups were identified, namely UKALL high hyperdiploid poor risk patients with MRD of 0·03% or greater or slow early responders. Patients with a UKALL high hyperdiploid good risk profile and MRD less than 0·03% had an excellent outcome. Hence the overall effect of MRD among UKALL high hyperdiploid good risk patients was borderline. Many protocols now classify patients with MRD of 5% or greater as refractory and treat them as very high risk.^26^ In this study, very few patients with a high hyperdiploid good risk profile had MRD of 5% or greater and their relapse rate was high, so these patients should be treated as refractory like other patients with MRD of 5% or greater. Further delineation of the high hyperdiploid good risk profile by MRD had a marginal effect in terms of the main endpoints. However, there could be some scenarios where such delineation is required (eg, some treatment reductions), and in such scenarios 0·03% would be the most appropriate threshold.

The UKALL high hyperdiploid profile builds on much of the previous evidence of outcome heterogeneity in patients with high hyperdiploid acute lymphoblastic leukaemia. We previously reported that +18 was associated with a good outcome.^15^ Additionally, +17 is part of the triple trisomy used by the COG to define low risk high hyperdiploid acute lymphoblastic leukaemia.^27^ Both +5 and +20 have been associated with a poor outcome in paediatric and adult patients with high hyperdiploid acute lymphoblastic leukaemia.^6^, ^28^ The four chromosomes driving our profile map to three of the four high hyperdiploid chromosome sets proposed by Heerema and colleagues;^17^ hence, our profile might represent a simplification of their model. One hypothesis to explain the good outcome of patients with high hyperdiploidy is that high hyperdiploid cells preferentially take up methotrexate. Although the functional consequences of high hyperdiploidy itself remain to be fully elucidated, differential expression of genes on the trisomic chromosomes is the dominant theory.^29^ This idea would support the concept that specific patterns of trisomies could influence the response to therapy and hence prognosis. Further studies are needed to understand the functional consequences of the different combinations of trisomies within high hyperdiploidy.

There are a few limitations to our study. The extensive analysis in this study required access to two large datasets. Hence, our discovery dataset comes from the pre-MRD, era but this fact was mitigated to some degree by using a contemporary dataset from a MRD-based protocol. Also, we had to rely on cytogenetic data derived from classical karyotyping. Bone marrow samples can sometimes produce poor quality metaphases, which makes identifying chromosomes, particularly smaller chromosomes, challenging. However, comparing the distribution of gained chromosomes in high hyperdiploidy determined by cytogenetics and single nucleotide polymorphism (SNP) array revealed no differences.^15^, ^29^ Additionally, 53% of patients had both +17 and +18 and our profile automatically assigns these patients to the good group. Patients with either +17 or +18 are also assigned to the good risk group unless they have +5 or +20. Our data suggest that +5 and +20 are rare and SNP array studies confirm this observation.^29^ Therefore, despite the limitations of using cytogenetic data, we are confident that the misclassification of patients in this study is low. Moving forward, widespread use of SNP arrays during the diagnosis of acute lymphoblastic leukaemia will ensure the accurate identification of all gained chromosomes in patients with high hyperdiploidy. The application of this novel profile to additional datasets will provide further evidence regarding its robustness and valuable information regarding its clinical usefulness across different treatment protocols.

The UKALL-HeH profile is simple to compute from a full karyotype or, preferably, SNP array profile. Although we acknowledge the difficulty of recognising the smaller chromosomes, our profile can also be readily determined using centromere or locus-specific fluorescence in-situ hybridisation in the event of a normal, failed, or incomplete karyotype. Although DNA index can be used to determine ploidy, it cannot ascertain which chromosomes are gained or lost, so the UKALL high hyperdiploid profile cannot be determined by DNA index. The most widely used trisomy profile in patients with high hyperdiploid acute lymphoblastic leukaemia is the tiple trisomy developed by the COG. With respect to outcome metrics, the UKALL high hyperdiploid profile matches or outperforms triple trisomy. The major advantage of our new profile is that the good risk group captures a much larger group of patients with a low risk of relapse, while defining a smaller poor risk group that is more likely to have high-risk relapses. Therefore, the profile offers advantages in the clinic in term of risk stratification.

In conclusion, by performing, to our knowledge, the most comprehensive analysis to date of risk factors in childhood high hyperdiploid acute lymphoblastic leukaemia, we identified a robust and clinically useful profile on the basis of the trisomic status of four chromosomes. It is reassuring that the constituent trisomies (+5, +17, +18, and +20) have all previously been proposed as risk factors in high hyperdiploidy and provide a framework for further investigations to elucidate precisely which genes are determining treatment response. Our risk profile outperformed previously reported risk profiles in high hyperdiploidy in terms of prediction accuracy. The prognostic effect of this profile is independent of MRD but can be refined by its integration, supporting the concept that integrating key risk features in childhood acute lymphoblastic leukaemia will improve risk stratification. If high hyperdiploidy is to be included in clinical trial stratification criteria to identify patients eligible for treatment de-intensification, it is crucial to remove high hyperdiploid poor risk patients from this group, as they have an intermediate prognosis and should be considered for treatment intensification. Therefore, we propose that the UKALL high hyperdiploid good risk profile is superior in defining good risk high hyperdiploidy compared with counting chromosomes.

## Data sharing

The National Cancer Research Institute Children's Cancer and Leukaemia Group Leukaemia Subgroup will consider data sharing requests from researchers investigating questions regarding the biology and treatment of acute lymphoblastic leukaemia. Data, including individual patient data, and study details will be released if the project is deemed pertinent. Initial requests should be directed to Prof Anthony Moorman (anthony.moorman@newcastle.ac.uk).

## Declaration of interests

We declare no competing interests.
