
JOURNAL INFORMATION
==============================
NLM Title Abbreviation: Lancet Haematol
Journal ID: Lancet Haematol
NLM Journal ID: 101643584

The Lancet. Haematology

EISSN: 2352-3026

ARTICLE INFORMATION
==============================
PMCID: PMC8610814
PMID: 34774148
DOI: 10.1016/S2352-3026(21)00340-9
Article ID: S2352-3026(21)00340-9
Article version: 1
Subjects: Corrections

Correction to Lancet Haematol 2021; 8: e828–39

Publication date: 2021 Dec
Electronic publication date: 2021 Nov 10
Volume: 8
Issue: 12
First page: e874
Last page: e874
Copyright: Crown Copyright © 2021 Published by Elsevier Ltd.
Copyright year: 2021
Copyright holder: Elsevier Ltd
License: This is an open access article under the CC BY license (http://creativecommons.org/licenses/by/4.0/).
License URL: https://creativecommons.org/licenses/by/4.0/

Erratum for: Defining low-risk high hyperdiploidy in patients with paediatric acute lymphoblastic leukaemia: a retrospective analysis of data from the UKALL97/99 and UKALL2003 clinical trials 8 11 26 10 2021 e828 e839 The Lancet. Haematology 10.1016/S2352-3026(21)00304-5 PMC8567211 34715050

==============================
Enshaei A, Vora A, Harrison CJ, Moppett J, Moorman AV. Defining low-risk high hyperdiploidy in patients with paediatric acute lymphoblastic leukaemia: a retrospective analysis of data from the UKALL97/99 and UKALL2003 clinical trials. Lancet Haematol 2021; 8: e828–39—In this Article, the second sentence of the Methods in the Summary should have read “…UKALL2003 recruited children and young adults aged 1–24 years between Oct 1, 2003, and June 30, 2011…” and the first sentence of the Study design and participants section of the methods should have read “…or UKALL2003 (between Oct 1, 2003, and June 30, 2011…”. Also, the data in the “One delayed intensification block” and “Two delayed intensification block” rows of table 2 have been corrected. These corrections have been made as of Nov 10, 2021.

