# Supplementary material for: Defining low-risk high hyperdiploidy in patients with paediatric acute lymphoblastic leukaemia: a retrospective analysis of data from the UKALL97/99 and UKALL2003 clinical trials
Source: Lancet Haematol. 2021 Oct 26;8(11):e828–39. doi: 10.1016/S2352-3026(21)00304-5 (PMC8567211; doi:10.1016/S2352-3026(21)00304-5)
Supplement: Supplementary appendix [file mmc1.pdf]

THE LANCET
Haematology

Supplementary appendix

This appendix formed part of the original submission and has been peer reviewed. We post it as supplied by the authors.

Supplement to: Enshaei A, Vora A, Harrison CJ, et al. Defining low-risk high hyperdiploidy in patients with paediatric acute lymphoblastic leukaemia: a retrospective analysis of data from the UKALL97/99 and UKALL2003 clinical trials. *Lancet Haematol* 2021; **8:** e828–39.

Supplementary Figure 1: Discovery and evaluation of novel HeH-ALL sub-group

A) Each dot represents a specific combination of one, two, three, four, five and six trisomies which were significantly linked to outcome (n=600). Combinations with fewer than 10 patients were excluded. The y axis gives the C-Index for each significant combination (n>10 and adjusted p-value <0.05) and the box and whisker plot represents the mean, interquartile range and range for the C-index. B) Five significant chromosomes that were identified using univariate analysis were used to develop multivariate model. The goodness of the fit of the models were compared using likelihood ratio test. * Likelihood-ratio test

Supplementary Figure 2: Clinical applicability of the novel subgroup.

A) Area under curve comparing novel risk group to previously published risk profiles. This analysis indicates that prediction accuracy of Novel risk (0.66) is higher than any other established risk profiles. TT (0.60), DT (0.58), +18 (0.63), +5 (0.58). B) Comparison of C-index for cox regression models of three models: (1) MRD (Red), (2) MRD and triple trisomy (Green) and (3) MRD and novel (Blue) risk for 500 bootstrapped samples. Results indicate a significant improvement by adding the new risk profile and MRD in same model. This analysis suggests that combination of TT and MRD preforms no better compare to MRD alone.

Supplementary Figure 3: Novel subgroups identifies clinically relevant sub-groups within MRD negative patients.

(A-C) MRD negative, (D-E) MRD positive, (A,D) Event-free survival, (B,E) Relapse rate, (C,E) Overall survival; MRD positive (≥0.03%) MRD negative (<0.03%)

Supplementary Figure 4: Univariate cox models for individual trisomies in the UKALL2003 cohorts.

Hazard ratio and 95% confidence intervals for event free survival (EFS), relapse rate (RR) and overall survival (OS) comparing patients with and without each individual trisomy. * denotes the adjusted p-value of <0.05

Supplementary Figure 5: MRD distribution for patients with specific trisomies.

τ(MRD) was defined as the negative natural log of the absolute post induction MRD level with undetectable MRD being assigned values of 1x10-6 as previously described (*J Clin Oncol*. 2018;36(1):34-43.) Patients with trisomy 10, 11, 12, 18 and 22 had significantly lower MRD whereas patients with trisomy 9 or gain of X had significantly higher levels of MRD.

Supplementary Figure 6: Correlation Coefficient of chromosome gains.

This plot illustrates the correlation coefficient between sets of chromosome gain for 725 HeH-ALL patients who were treated on UKALL2003 trial. This plots highlights the distinct clusters of the gains that supports the previous research by Heerema et al. Briefly, these clusters are: 21, X, 14, 6, 18, 4, 17, 10 (cluster I) –8, 5, 11, 12 (cluster II) –2, 3, 9, 16, 22 (cluster III) – 1, 7, 13, 15, 19, 20 (cluster IV)- Y (cluster V). Any correlation less than 0.1 is excluded. Blue denotes positive correlation and red negative correlation. The thickness of the lines relates to the size of the correlation.

Supplementary Figure 7: Optimum number of trisomy(s) using Mallows' Cp statistic.

The model with the lowest value of the Mallows' Cp is the most precise model therefore model with combinations of 4 chromosome is the best model in terms of predicting the outcome.

Supplementary Figure 8: Independent validation of the optimum combination of 5, 17, 18 and 20

A) Bayesian information criterion (BIC) is a model selection tool which estimates model performance. This method assigns a BIC score to the whole set of candidate combination of gains. The model with the smallest BIC score over the complete set of possible models was deemed the least complex and fittest model and better prediction. Here the fittest model based on four chromosomes (with the lower value of BIC highlighted in red) was identified as combination of chromosome 18, 20, 5 and 17. .
B) Development of correlation coefficient network of gained chromosomes and relapse indicates that only +18, +17, +20 , +11 and +5 have strong linear correlation with relapse. The thickness of the lines represents the correlation between nodes (the thicker the line the stronger

correlation). The colour of the line represent the direction of the correlation (blue: positive correlation, red: negative correlation). All correlations less than 0.1 are excluded. Blue denotes positive correlation and red negative correlation. The thickness of the lines relates to the size of the correlation

C) We generated all possible combinations of presence and absence (noted by "!") of four chromosomes. We plotted the coefficient against log of adjusted p-value. The significant good risk profiles on the left (green) and significant poor risk profiles on the right (red). All of the good risk profiles include +17 or +18 and lack +5 and +20. On the other hand all of the poor profiles include +5 or +20. Risk profiles with less than 10 cases were excluded.

Supplementary Figure 9: A graph illustrating the cumulative distribution of HeH cases treated on UKALL2003 by end of induction MRD, alongside the 10 year relapse risk for patients below (green line) and above (orange line) each threshold. The optimal threshold is determined by considering both the shift in relapse rate from one threshold to the next and the size of the respective groups that this threshold would produce. Thus a threshold of 0.03% produces two groups with a size ratio of 73:27 and a doubling of the relapse rate.

Supplementary Figure 10: Sensitivity analysis for novel HeH risk groups indicates that the profile in significant in key UKALL2003 subgroups

(A) Event-free survival; (B) Relapse rate; (C) Overall survival

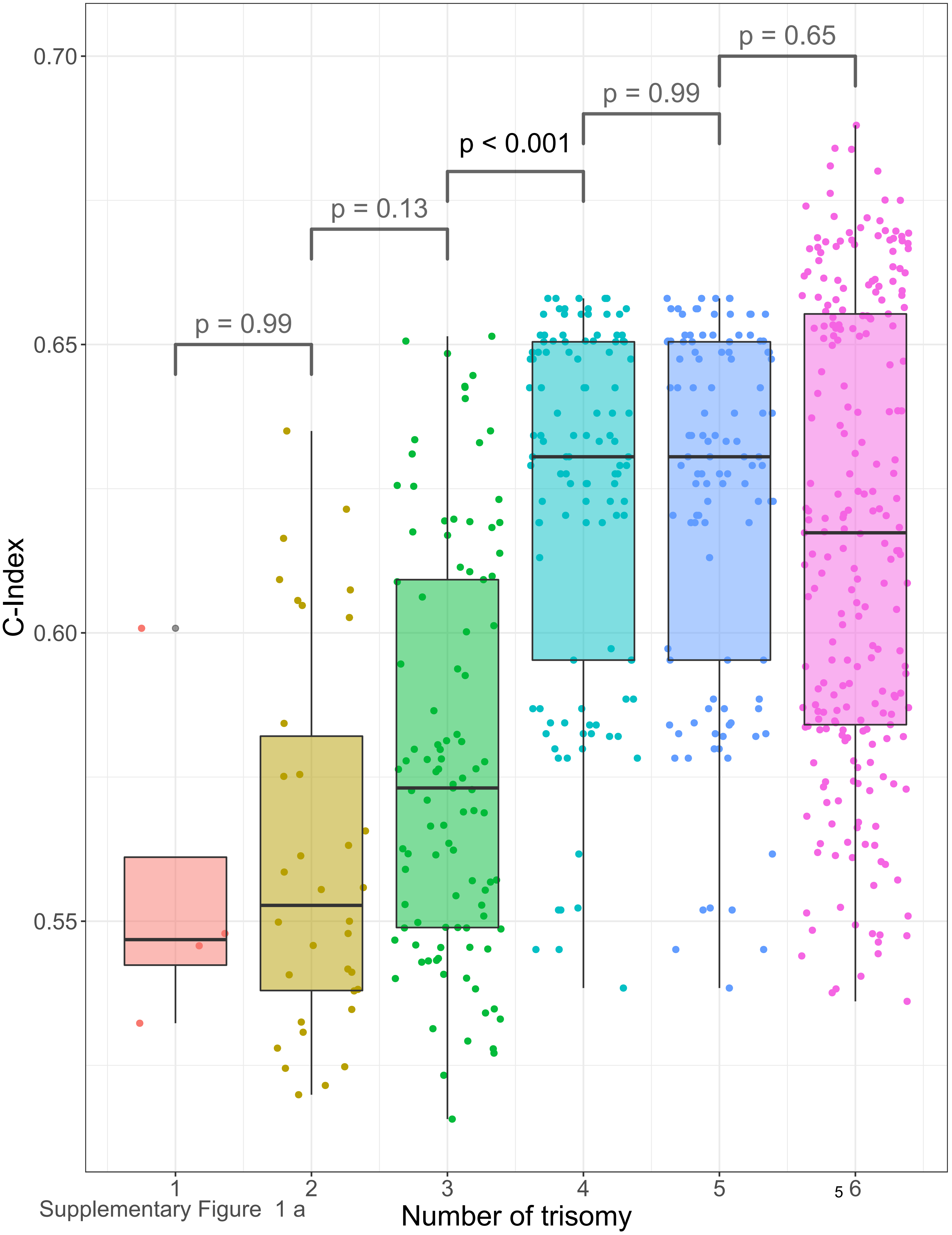

Supplementary Figure 1 a

5

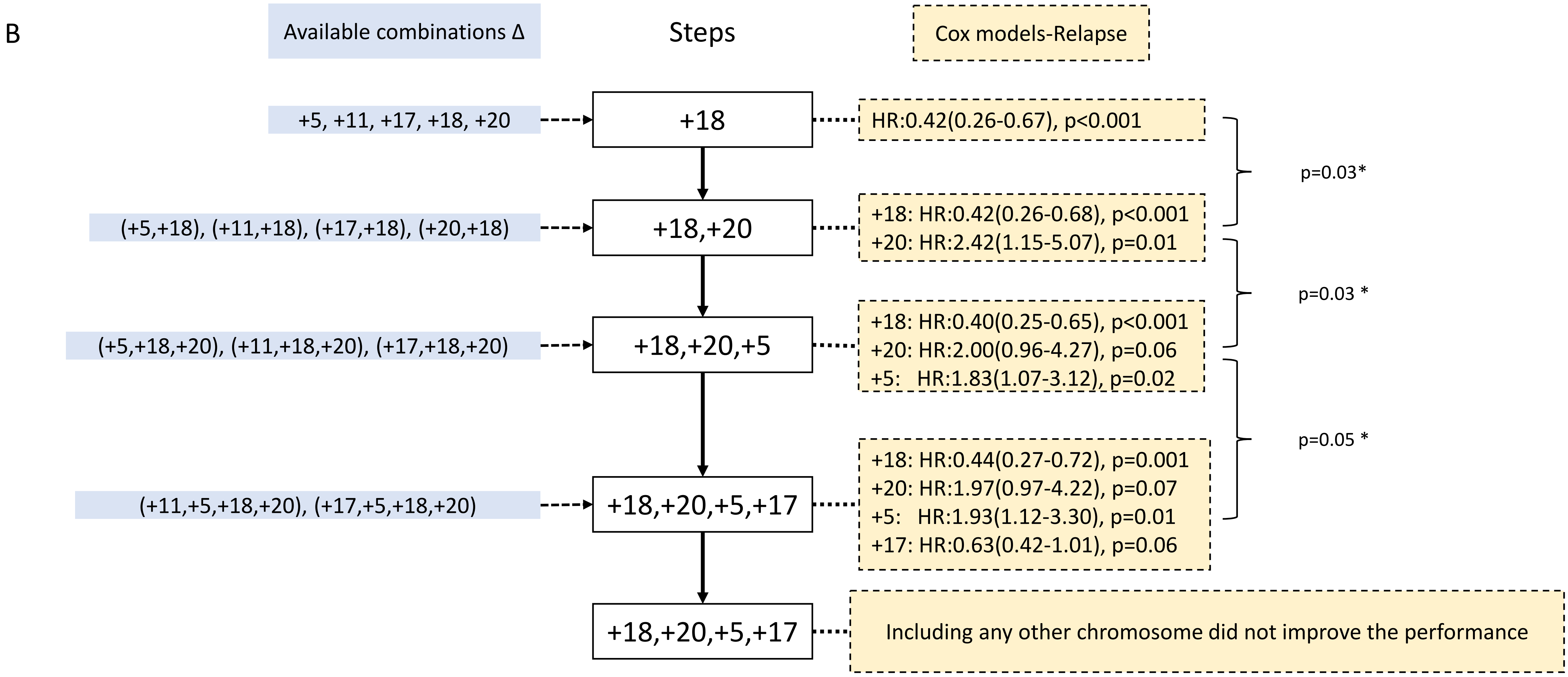

Supplementary Figure 1 b

A

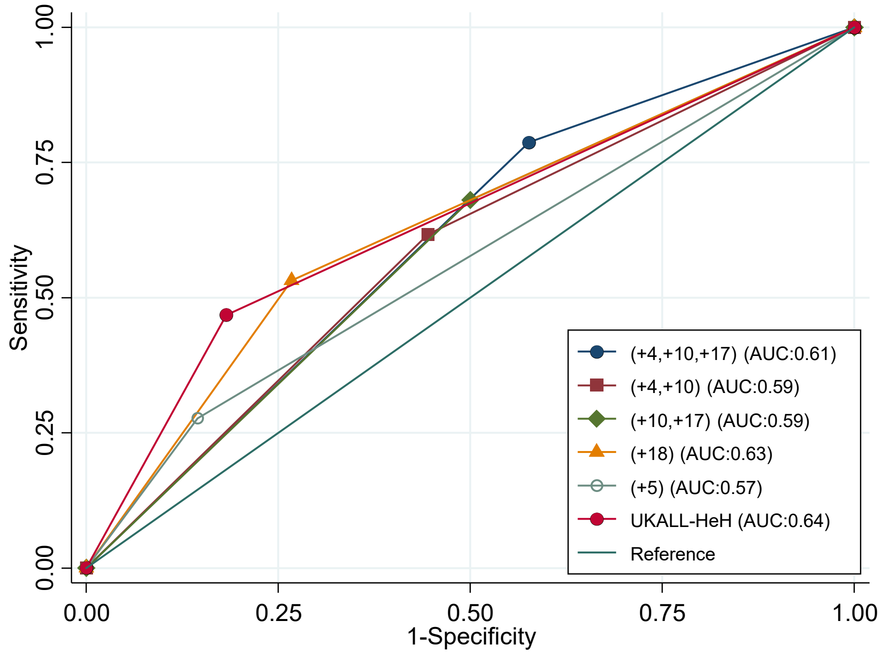

Supplementary Figure 2 a

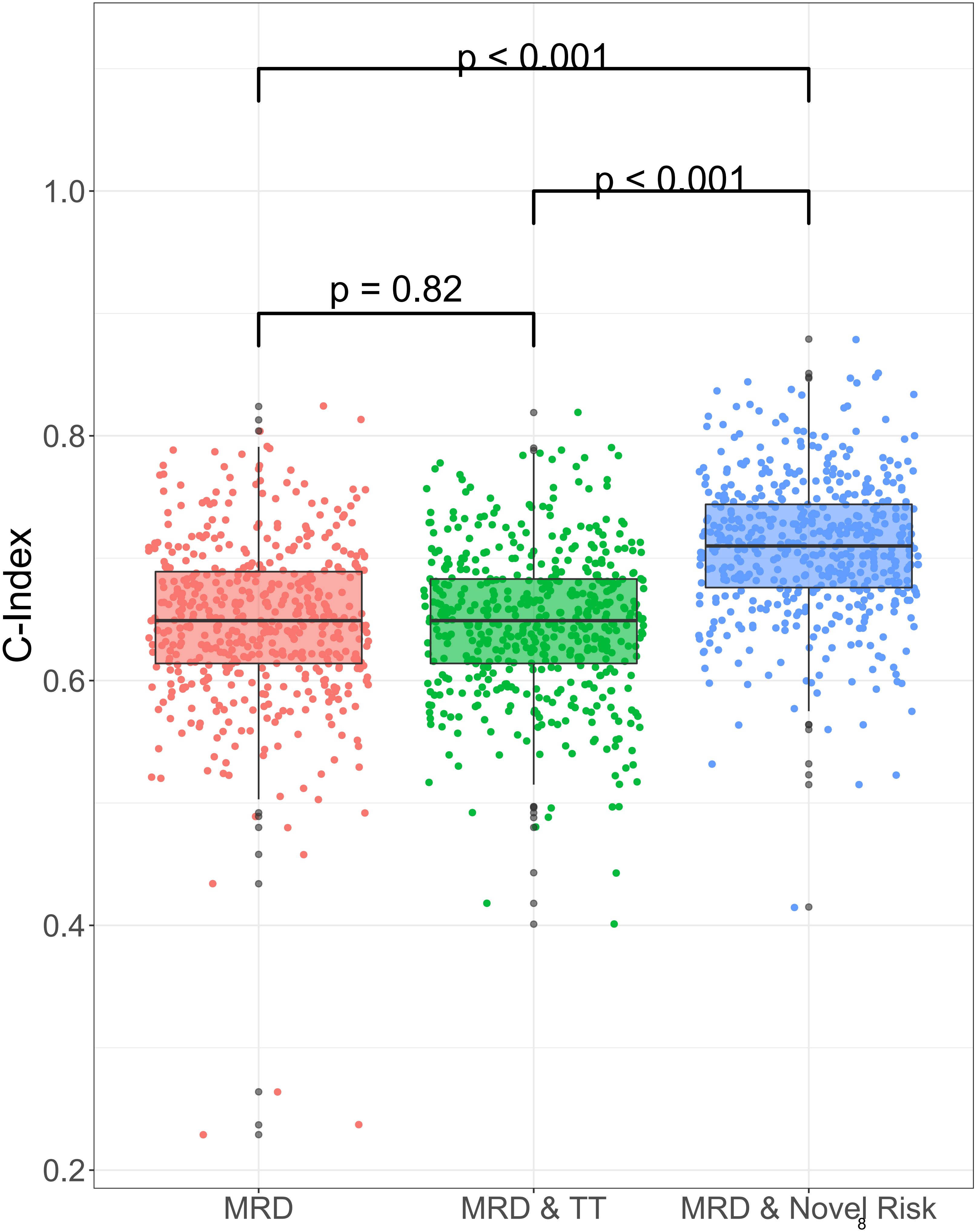

Supplementary Figure 2 b

Supplementary Figure 3

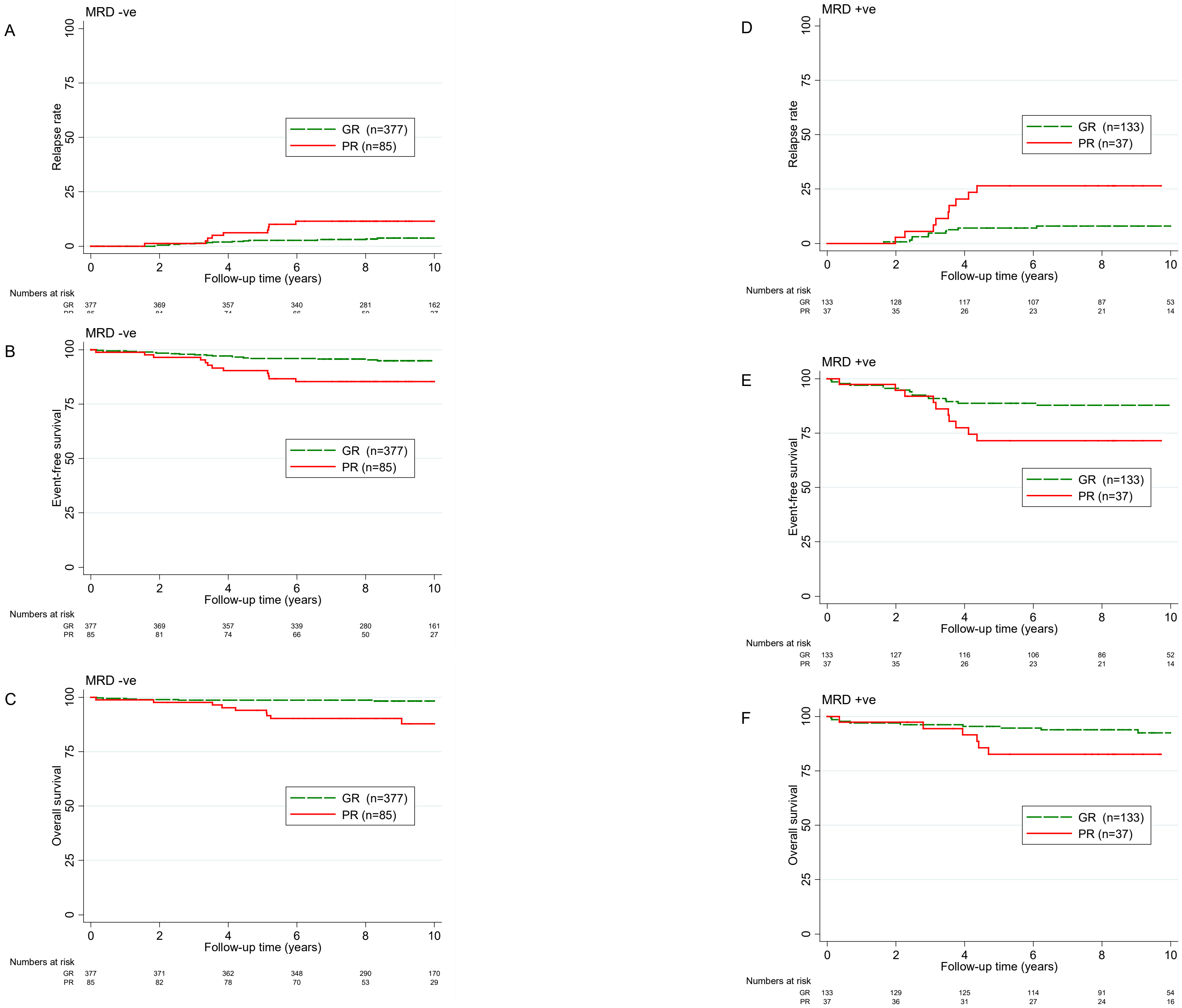

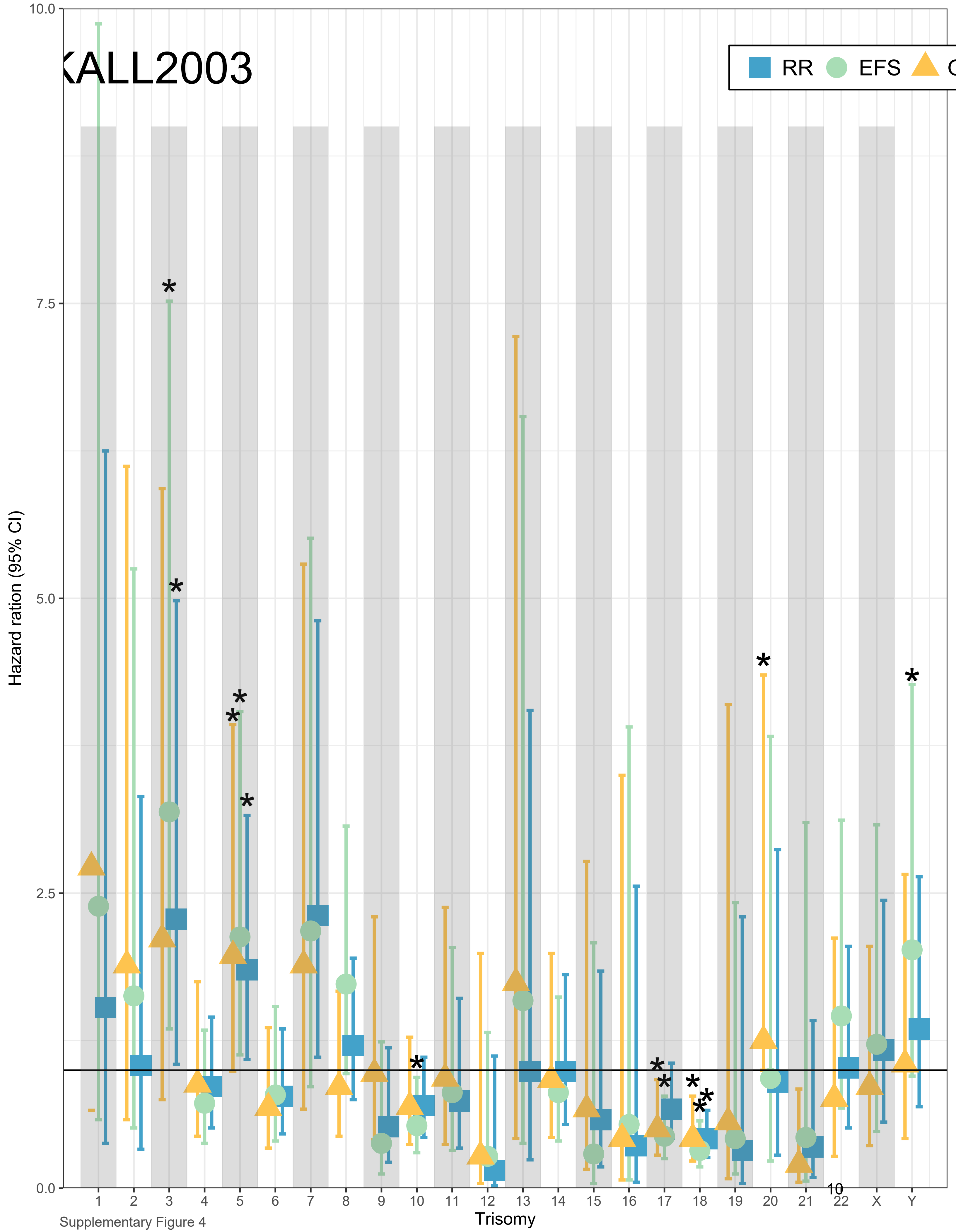

Supplementary Figure 4

Trisomy	1	2	3	4	5	6	7	8	9	10	11	12	13	14	15	16	17	18	19	20	21	22	X	Y
Number of patients	13	28	34	553	111	582	39	229	106	491	89	57	21	594	49	26	480	519	31	33	717	87	632	91

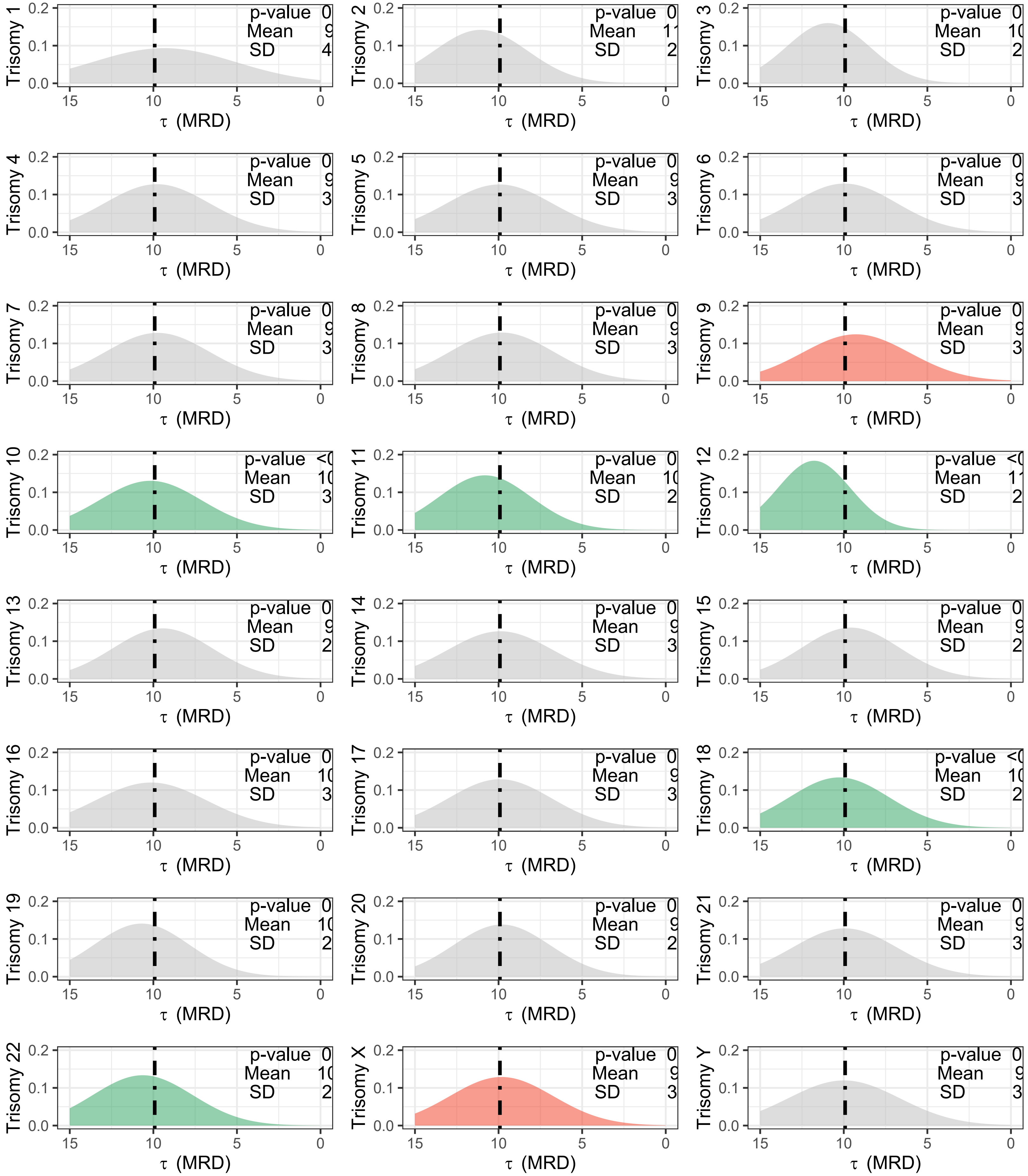

Supplementary Figure 5

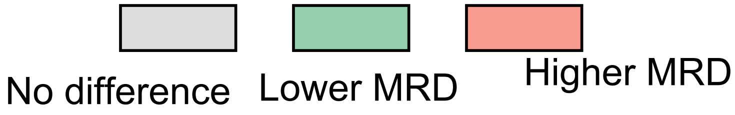

Supplementary Figure 6

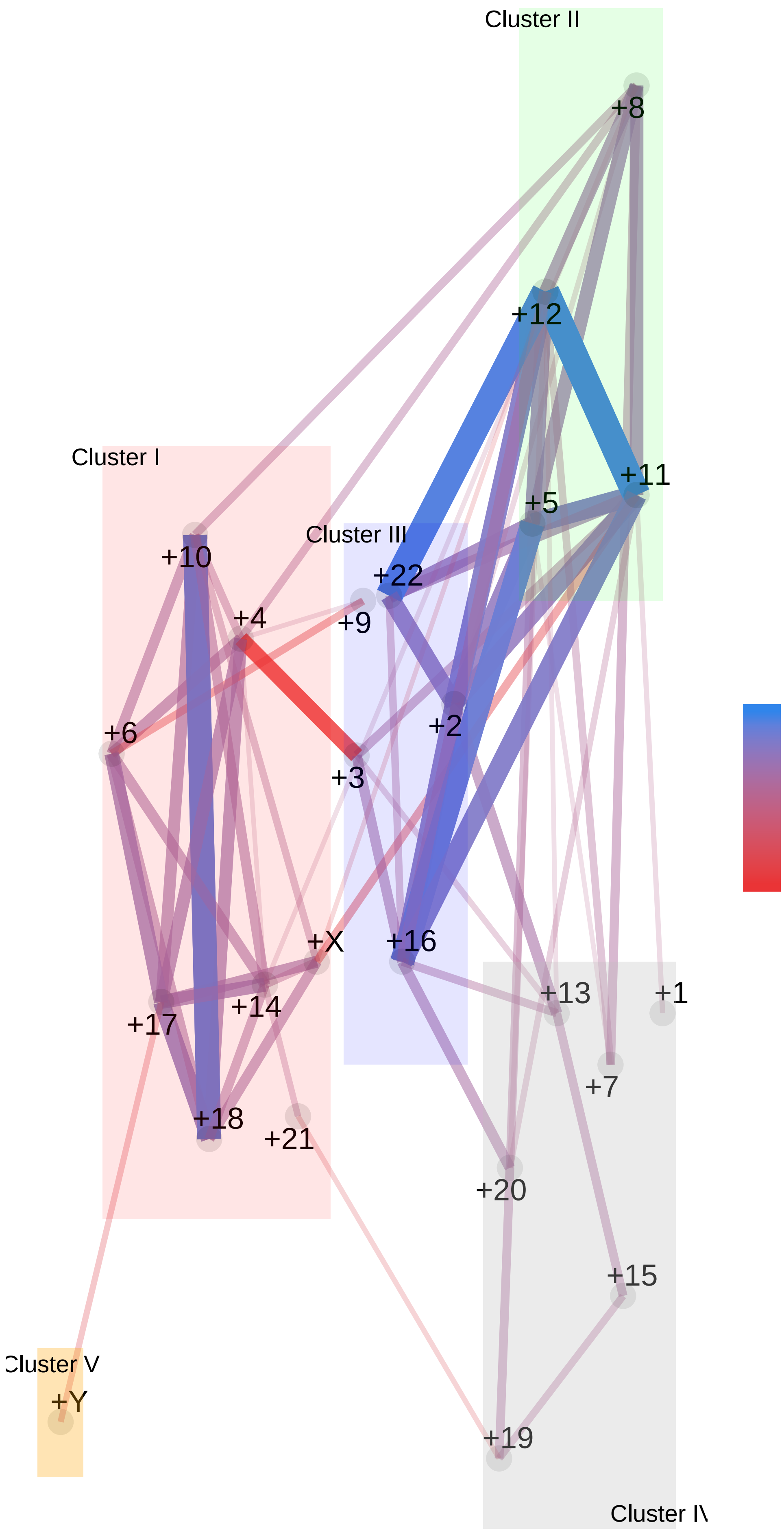

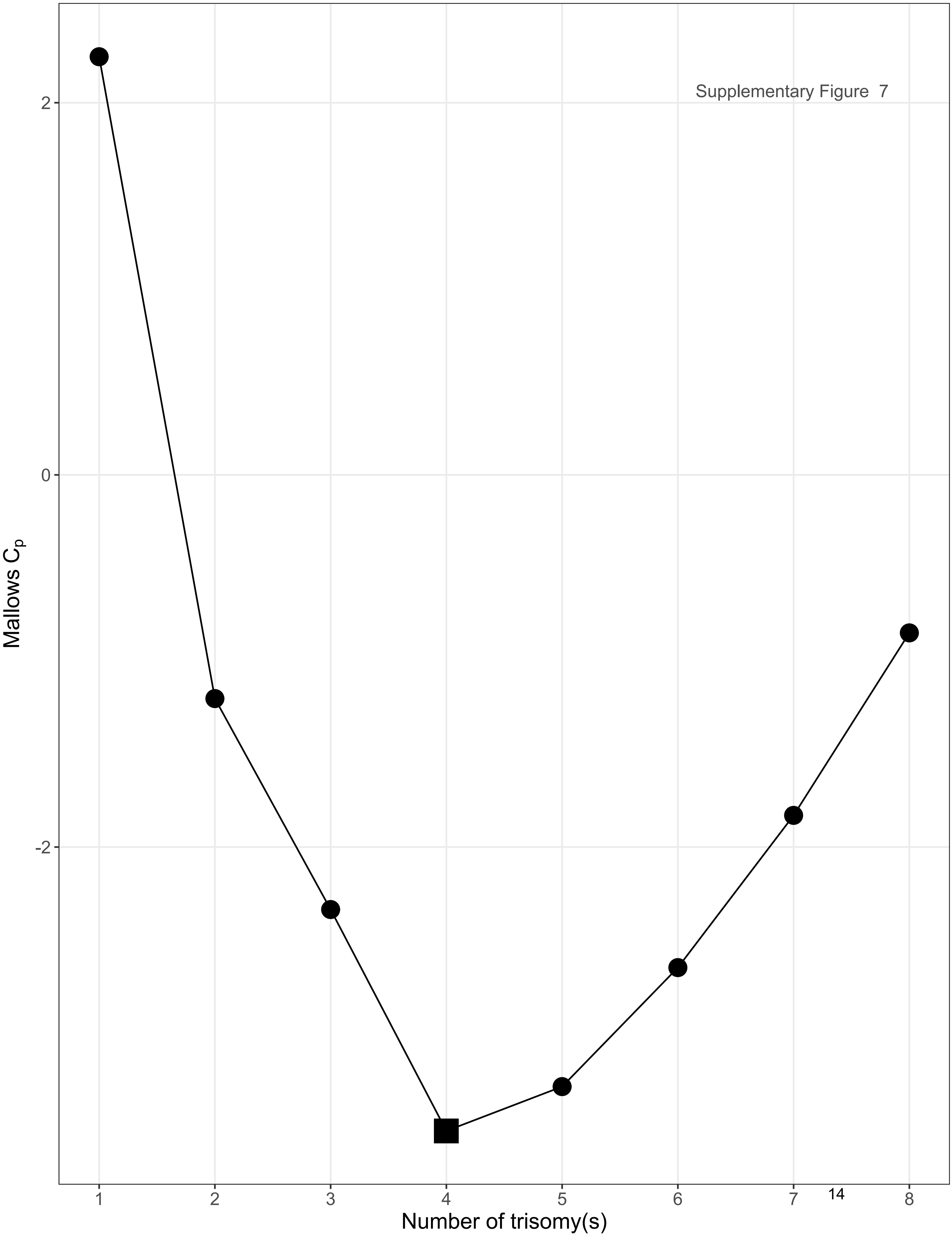

Supplementary Figure 7
Mallows C_p
2
0
-2
1
2
3
4
5
6
7
8
Number of trisomy(s)

A

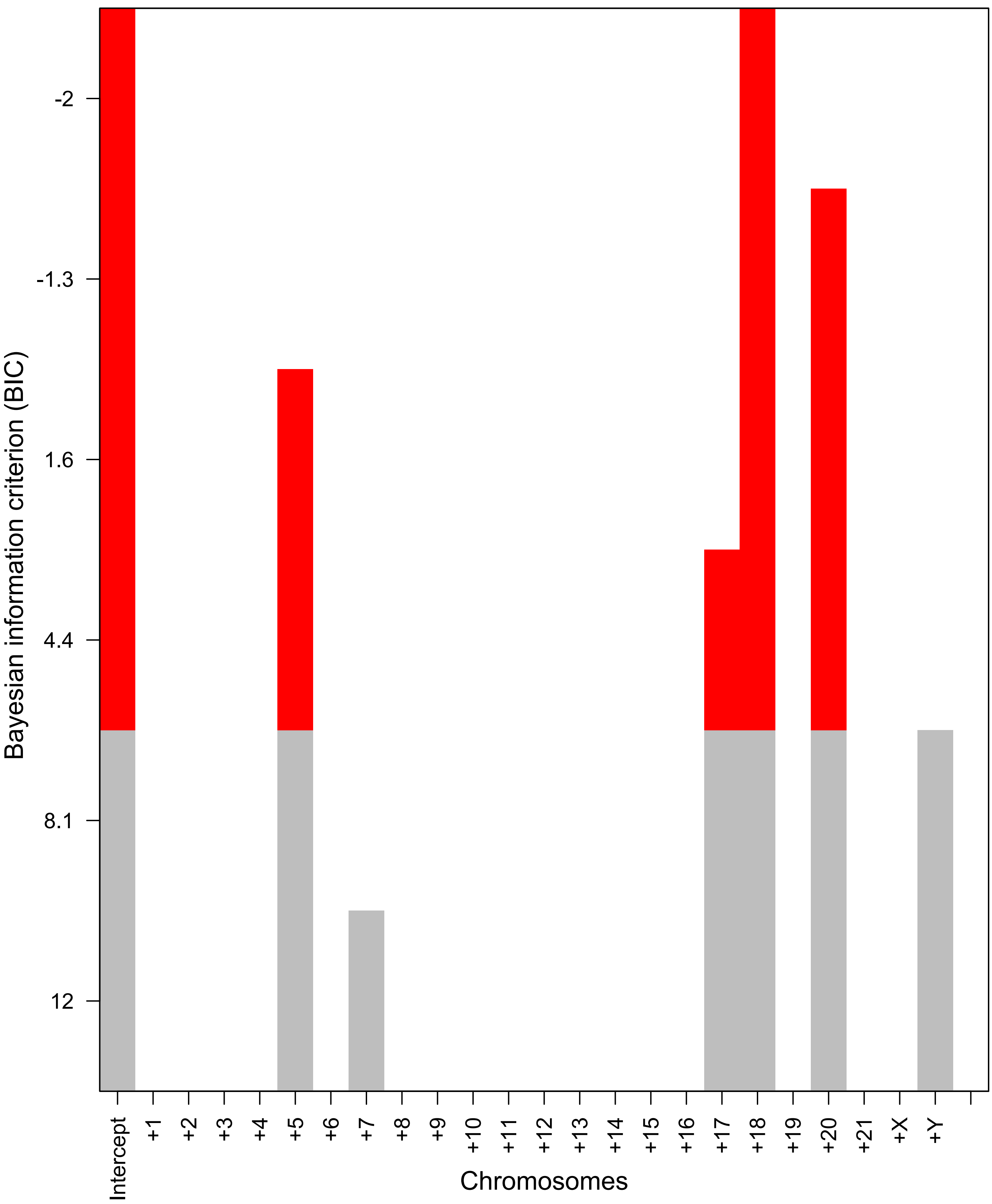

Supplementary Figure 8 a

B

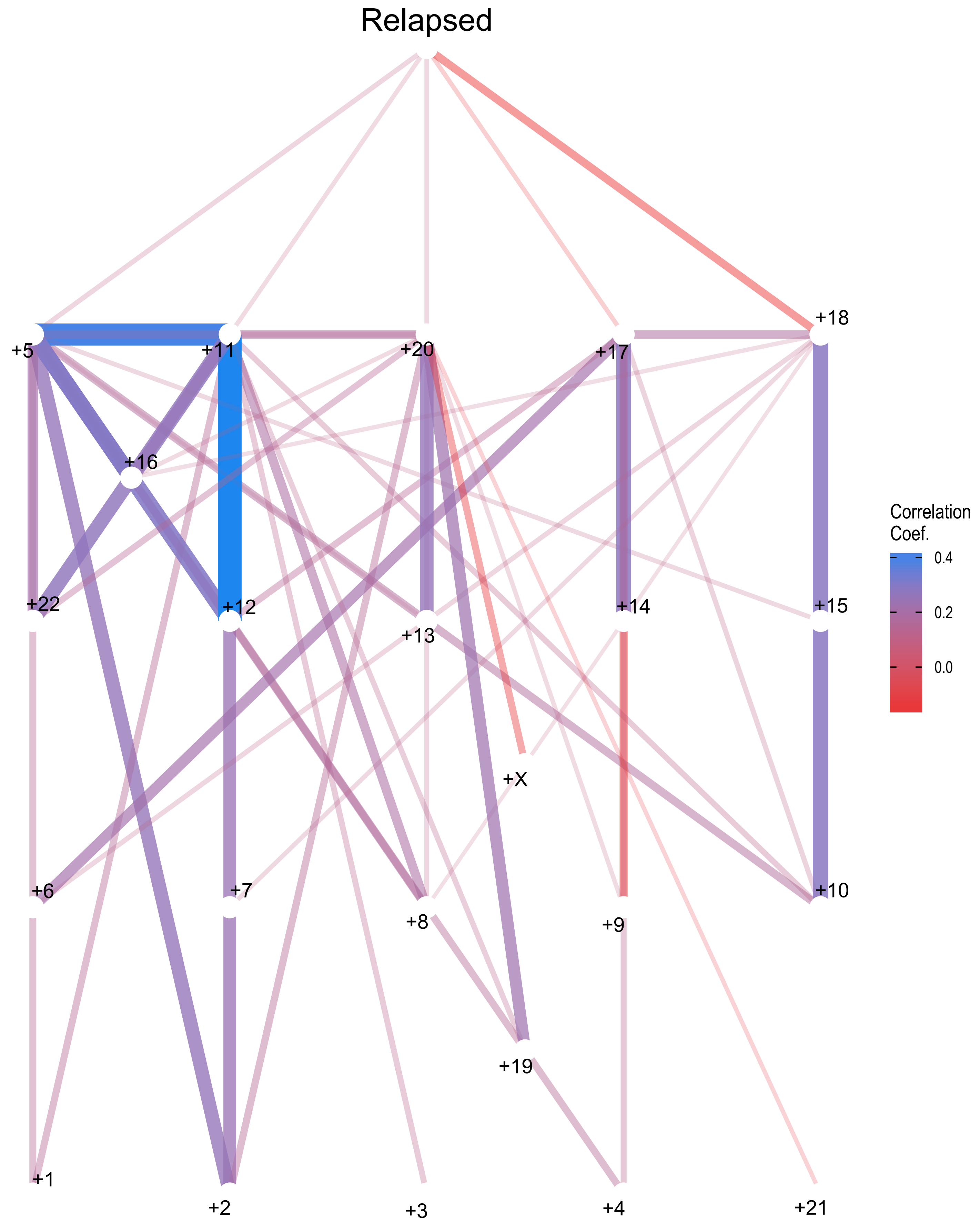

Supplementary Figure 8 b

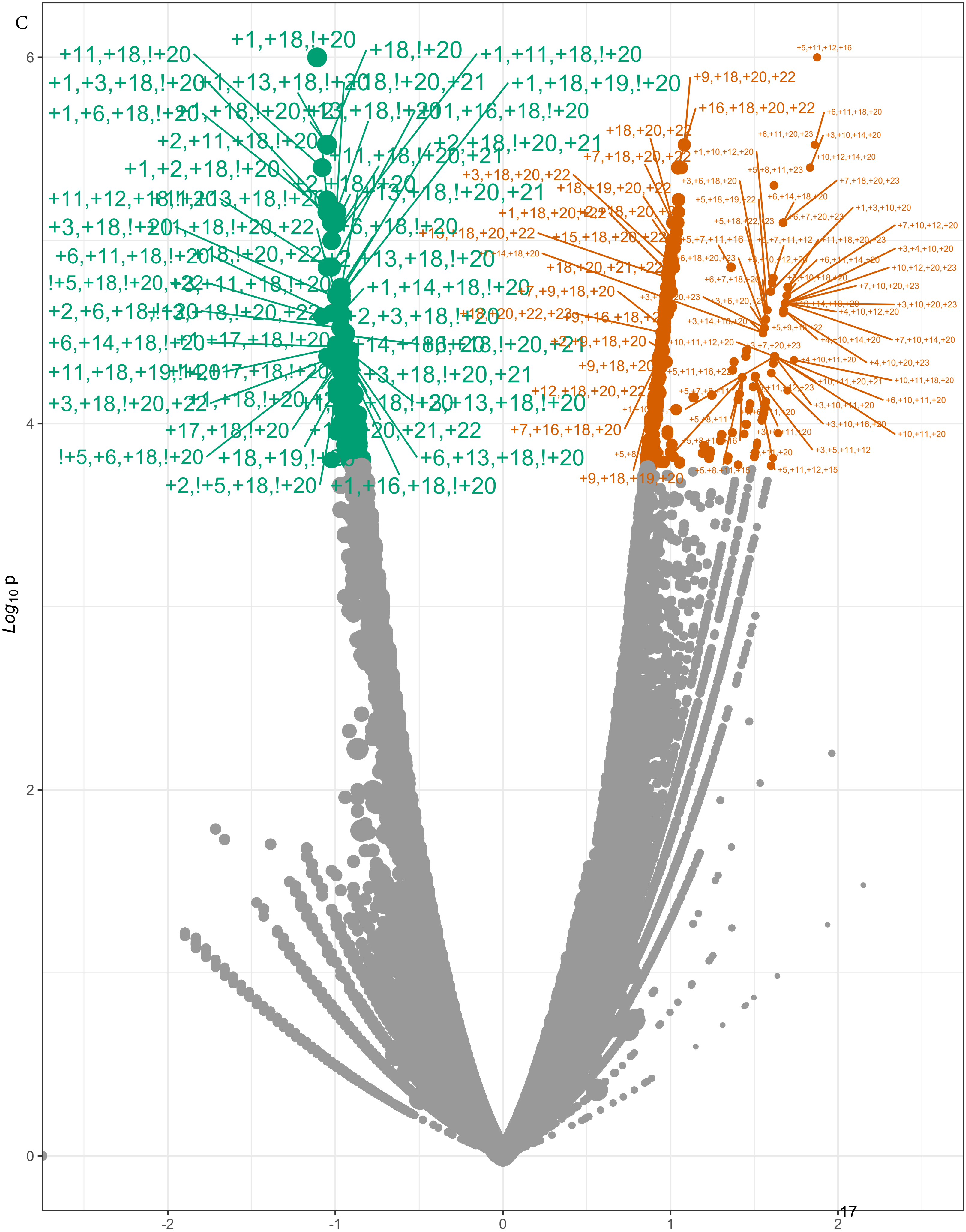

Supplementary Figure 8 c

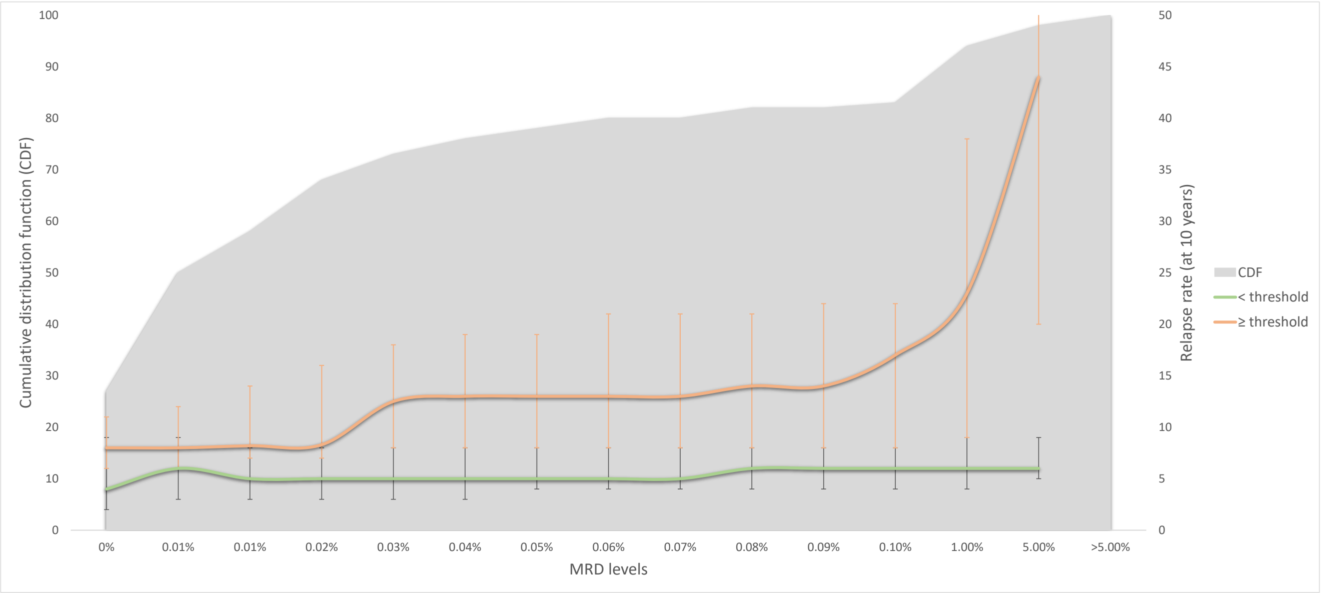

Supplementary Figure 9

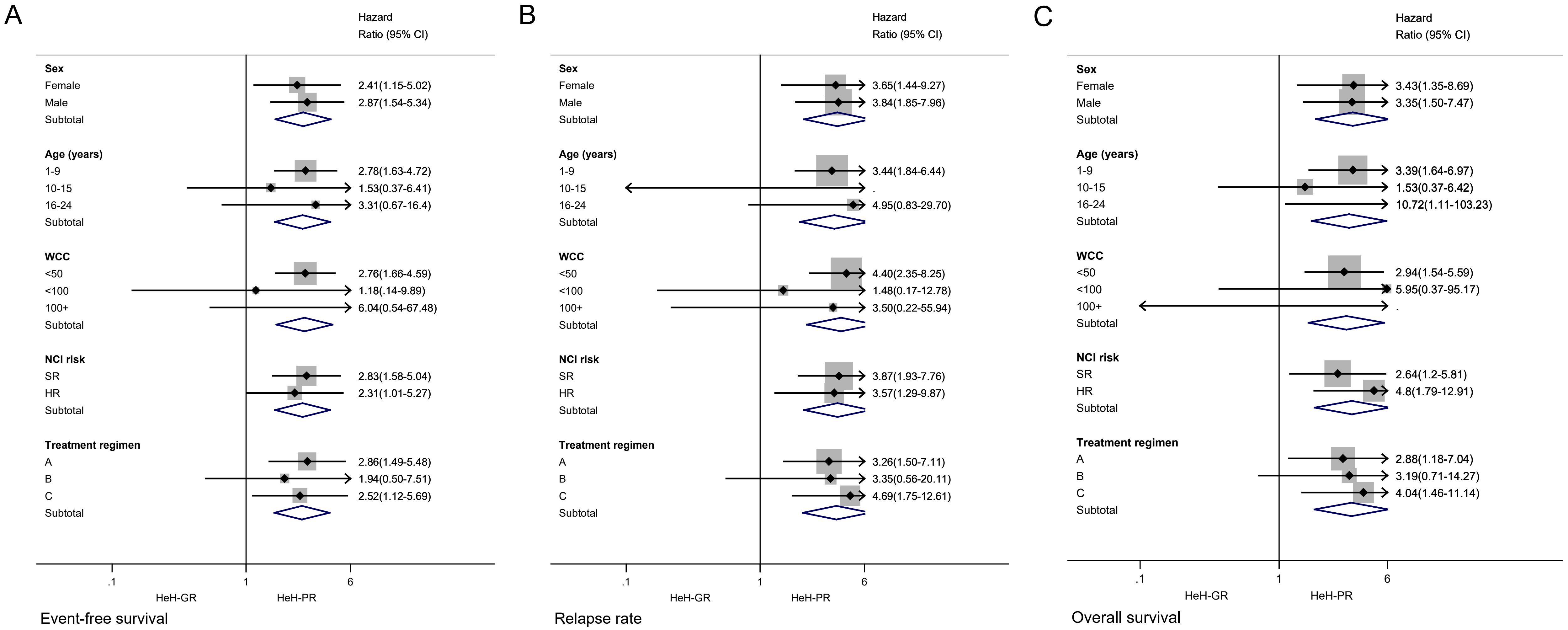

Supplementary Figure 10

Supplementary Table 1: Univariate analysis of UKALL2003 high hyperdiploid cases by previously published risk factors

		Hazard ratio (95% Confidence Interval), p-value		
	n (%)	Relapse rate	Event free survival	Overall survival
Modal chromosome number				
As a continuous variable	457	0.84 (0.70-1.01), 0.08	0.95 (0.82-1.08), 0.41	0.95 (0.79-1.14), 0.58
51-53 chromosomes	120 (20)	2.29 (1.13-4.65), 0.02	1.75 (0.97-3.15), 0.06	1.62 (0.73-3.61), 0.24
54-57 chromosomes	383 (64)	1	1	1
58-65 chromosomes	92 (16)	0.64 (0.19-2.18), 0.48	0.91 (0.40-2.06), 0.82	0.70 (0.21-2.38), 0.57
Trisomy profiles				
Double trisomy (+4,+10) v Remaining HeH cases	395 (54) v 330 (46)	0.51 (0.28-0.91), 0.02	0.74 (0.47-01.18), 0.2	0.84 (0.46-01.53), 0.56
Triple trisomy (+4,+10,+17) v Remaining HeH cases	299 (41) v 426 (59)	0.38 (0.19-0.77), 0.01	0.71 (0.44-01.16), 0.18	0.64 (0.33-01.24), 0.19
Age (years)				
As a continuous variable	725 (100)	1.04 (0.98-1.11), 0.19	1.04 (0.99-1.09), 0.15	1.08 (1.01-1.14), 0.02
Age ≥10 v Age<10	111 (15) v 614 (85)	1.02 (0.46-2.28), 0.96	1.39 (0.78-2.50), 0.26	2.35 (1.20-4.58), 0.01
White cell count (WCC) (x10^9/L)				
As a continuous variable*	725 (100)	1.22 (0.95-1.55), 0.12	1.18 (0.97-1.43), 0.11	1.03 (0.79-1.34), 0.81
WCC ≥ 50 v WCC<50	69 (10) v 656 (90)	2.04 (0.95-4.37), 0.07	1.59 (0.81-03.1), 0.18	1.03 (0.37-2.88), 0.96
End of induction Minimal Residual Disease (MRD)				

As a continuous variable [τ(MRD)]**	632 (100)	0.85 (0.77-0.93), <0.0001	0.85 (0.78-0.92), <0.0001	0.81 (0.73-0.91), <0.0001
MRD ≥0.01% v <0.01%	262 (41) v 370 (59)	2.26 (1.25-4.08), 0.007	2.30 (1.11-4.75), 0.02	3.48 (1.44-8.39), 0.005
MRD ≥0.03% v <0.03%	170 (27) v 462 (73)	2.43 (1.32-4.46), <0.0001	2.44 (1.45-4.11),	2.81 (1.37-5.75), <0.0001
Heerema et al clusters				
Cluster I	241 (33)	1	1	1
Cluster II v Cluster I	130 (18)	3.16 (1.32-7.54), 0.009	1.72 (0.90-3.27), 0.10	1.25 (0.53-2.93), 0.60
Cluster III v Cluster I	135 (19)	1.71 (0.64-4.56), 0.28	1.09 (0.53-2.24), 0.82	1.07 (0.44-2.57), 0.88
Cluster IV v Cluster I	128 (18)	1.36 (0.47-3.92), 0.57	1.06 (0.51-2.23), 0.87	0.84 (0.32-2.22), 0.73
Cluster V v Cluster I	91 (13)	3.64 (1.46-9.05), 0.005	1.66 (0.81-3.42), 0.17	1.19 (0.45-3.13), 0.72
Modal number & UK-HeH risk				
UK-HeH-PR		6.19 (2.81-13.66), <0.0001	4.45 (2.37-8.37), <0.0001	5.27(2.27-12.24), 0.001
Modal chromosome number		0.96 (0.81-1.12), 0.59	1.02 (0.90-1.15), 0.80	1.03(0.88-1.21), 0.73

Abbreviations and notes: * [ln(WCC)+1]; ** τ(MRD) was defined as the negative natural log of the absolute post induction MRD level with undetectable MRD being assigned values of 1x10-6 as previously described (J Clin Oncol. 2018;36(1):34-43.)

In UKALL2003, minimal residual disease (MRD) was evaluated by real-time quantitative polymerase chain reaction (PCR) analysis of immunoglobulin and T-cell receptor gene rearrangements as defined by the European MRD Study Group.[1] Patients were classified into 1 of 3 groups based on their MRD results: (1) LR (patients with undetectable MRD at the end of induction [day 29] or patients with low-level MRD [<0.01%] at day 29 and undetectable MRD at the start of interim maintenance); (2) HR (patients with MRD >0.01% at day 29); or (3) indeterminate group (patients with no MRD results due to no or poor sample and patients with persistent low-level disease [<0.01% MRD] before the start of interim maintenance).[2] Full details of the treatment regimens have been published.[2,3] Briefly, in ALL99 and UKALL2003, patients were assigned to regimen A or B based on whether they were National Cancer Institute (NCI) standard (<10 years old and WCC <50 × 10^9/L) or HR (≥10 years old or WCC >50 × 10^9/L), respectively. Regimen A comprised a 3-drug induction (vincristine, steroids, and asparaginase) followed by consolidation (daily mercaptopurine and weekly intrathecal methotrexate), central nervous system–directed therapy, interim maintenance (daily mercaptopurine, weekly methotrexate, monthly vincristine, and steroid pulses), delayed intensification (asparaginase, vincristine, dexamethasone, and doxorubicin), and continuing therapy (oral mercaptopurine and methotrexate, monthly vincristine and steroid pulses, and intrathecal methotrexate every 3 months). Regimen B patients additionally received daunorubicin during induction and Berlin-Frankfurt-Münster consolidation (4 weeks of cyclophosphamide and cytarabine). Regimen C patients received an additional 4 doses of vincristine and 2 doses of PEGylated asparaginase during Berlin-Frankfurt-Münster consolidation. Furthermore, regimen C patients received escalating doses of intravenous methotrexate without folinic acid rescue and vincristine and PEGylated asparaginase as interim maintenance (Capizzi maintainance).
Cytogenetic and fluorescence in situ hybridization testing was performed on pretreatment bone marrow samples by member laboratories of the UK Cancer Cytogenetics Group or centrally by the Leukaemia Research Cytogenetics Group, and results were reported using established nomenclature and definitions.[4] Multiplex ligation-dependent probe amplification (MLPA) was performed on DNA extracted either directly from pretreatment bone marrow samples or from fixed cell suspensions. The tested cohorts were representative in terms of sex, age, and outcome but were marginally enriched for patients with higher WCC and HR cytogenetics (supplemental Table 1). The SALSA MLPA kit P335 (MRC Holland, Amsterdam, The Netherlands), which included probes for *IKZF1*, *CDKN2A/B*, *PAX5*, *EBF1*, *ETV6*, *BTG1*, *RB1*, and PAR1 (*CSF2RA/IL3RA/CRLF2*), was used to identify CNAs in these genes.[5] Previous studies have demonstrated that MLPA can accurately detect deletions in all these genes, which are present in more than 20% to 30% of cells.[5,6] A detailed description and breakdown of each CNA and the correlation with specific chromosomal abnormalities for all the patients in these 2 cohorts has been published. [5]

1. van der Velden VH, Cazzaniga G, Schrauder A, et al. European Study Group on MRD detection in ALL (ESG-MRD-ALL)Analysis of minimal residual disease by Ig/TCR gene rearrangements: guidelines for interpretation of real-time quantitative PCR data., Leukemia, 2007, vol. 21 4(pg. 604-611)
2. Vora A, Goulden N, Wade R, et al. A randomised trial of treatment reduction for children and young adults with minimal residual disease defined low risk acute lymphoblastic leukaemia., Lancet Oncol, 2013, vol. 14 3(pg. 199-209)
3. Vora A, Mitchell CD, Lennard L, et al. Medical Research Council; National Cancer Research Network Childhood Leukaemia Working PartyToxicity and efficacy of 6-thioguanine versus 6-

mercaptopurine in childhood lymphoblastic leukaemia: a randomised trial., Lancet, 2006, vol. 368 9544(pg. 1339-1348)
4. Moorman AV, Ensor HM, Richards SM, et al. Prognostic effect of chromosomal abnormalities in childhood B-cell precursor acute lymphoblastic leukaemia: results from the UK Medical Research Council ALL97/99 randomised trial., Lancet Oncol, 2010, vol. 11 5(pg. 429-438)
5. Schwab CJ, Chilton L, Morrison H, et al. Genes commonly deleted in childhood B-cell precursor ALL: association with cytogenetics and clinical features., Haematologica, 2013, vol. 98 7(pg. 1081-1088)
6. Schwab CJ, Jones LR, Morrison H, et al. Evaluation of multiplex ligation-dependent probe amplification as a method for the detection of copy number abnormalities in B-cell precursor acute lymphoblastic leukemia., Genes Chromosomes Cancer, 2010, vol. 49 12(pg. 1104-1113)

The optimum number of the gains

Best subset regression, another independent technique, was used to evaluate all the best-subset models. Briefly, we measured Mallows' C_p for each combination and the model with the lowest value was chosen as the fittest model. The result was that the combination of four gained chromosomes was selected as the best possible predictor of outcome (Sup Figure 4).

The optimum combination

This comprehensive analysis indicated that all significant good risk combinations included +18 or +17 and lacked +5 and +20 (Sup Figure5C). Moreover, every poor risk combination included either +5 or +20.

To verify the above finding, univariate analysis of the prognostic effect of the gain of particular chromosomes identified additional chromosomes 17 (HR: 0.57 (0.35-0.93), p-value: 0.025), 18 (HR: 0.42 (0.26-0.67), p-value< 0.001) had a reduced RR compared to those without. Patients with gain of chromosome 18 also had better EFS (0.43 (0.28-0.67), p-value<0.001) and OS (0.40 (0.22-0.73), p-value<0.001). In contrast, gain of chromosomes 5 (HR: 1.86 (1.11-3.11), p-value: 0.02), 11 (HR: 1.83 (1.04-3.21), p-value: 0.03) and 20 (HR: 2.53 (1.21-5.30), p-value: 0.01) had significantly higher RR (Figure 1B, Supplementary Figure 1). Furthermore, trisomy 20 patients had significantly worse EFS (HR: 2.33 (1.17-4.67), p-value: 0.02) and OS (HR: 3.49 (1.55-7.86), p-value: 0.003). We used these chromosomes in feed forward/backward manner to identify the optimum subset for outcome prediction, using likelihood ratio test log (Figure 1B). The combination of gain of chromosomes 5, 17, 18 and 20 were identified as the best possible combination for predicting outcome.

Bayesian information criterion (BIC) with Forward stepwise criteria was used for model selection (Sub Figure5A). This method assigns a BIC score to the whole set of candidate combination of gains. The model with the smallest BIC score over the complete set of possible models is the less complex and fitter model. The model with gains of chromosomes 18, 20, 5 and 17 had the lowest BIC score.

Next, we developed a network of correlation coefficient of chromosome gains and relapse (Sup Figure5B). The gain of five chromosomes (18, 17, 20, 5 and 11) had a significant correlation to relapse. Trisomies 17 and 18 were negative and trisomies 5, 11 and 20 were positively correlated. These results highlight the direct influence on outcome of gains of these five chromosomes. There were indirect paths from other trisomies to relapse, but they all included at least one of above gained chromosomes.

R packages

igraph (1.2.6), tidyverse (1.3.1), corrr (0.4.3), ggraph (2.0.5), ggplot2 (3.3.3), reshape2 (1.4.3) , ggsignif (0.6.2), leaps (3.1) and glmulti (1.0.8).
